# Hill-based dissimilarity indices and null models for analysis of microbial community assembly

**DOI:** 10.1186/s40168-020-00909-7

**Published:** 2020-09-11

**Authors:** Oskar Modin, Raquel Liébana, Soroush Saheb-Alam, Britt-Marie Wilén, Carolina Suarez, Malte Hermansson, Frank Persson

**Affiliations:** 1 Water Environment Technology, Architecture and Civil Engineering, Chalmers University of Technology, Gothenburg, Sweden; 2 Chemistry and Molecular Biology, University of Gothenburg, Gothenburg, Sweden

**Keywords:** Aerobic granular sludge, Amplicon sequencing, Beta diversity, Bioinformatics, Microbial ecology, Microbial fuel cell

## Abstract

**Background:**

High-throughput amplicon sequencing of marker genes, such as the 16S rRNA gene in Bacteria and Archaea, provides a wealth of information about the composition of microbial communities. To quantify differences between samples and draw conclusions about factors affecting community assembly, dissimilarity indices are typically used. However, results are subject to several biases, and data interpretation can be challenging. The Jaccard and Bray-Curtis indices, which are often used to quantify taxonomic dissimilarity, are not necessarily the most logical choices. Instead, we argue that Hill-based indices, which make it possible to systematically investigate the impact of relative abundance on dissimilarity, should be used for robust analysis of data. In combination with a null model, mechanisms of microbial community assembly can be analyzed. Here, we also introduce a new software, qdiv, which enables rapid calculations of Hill-based dissimilarity indices in combination with null models.

**Results:**

Using amplicon sequencing data from two experimental systems, aerobic granular sludge (AGS) reactors and microbial fuel cells (MFC), we show that the choice of dissimilarity index can have considerable impact on results and conclusions. High dissimilarity between replicates because of random sampling effects make incidence-based indices less suited for identifying differences between groups of samples. Determining a consensus table based on count tables generated with different bioinformatic pipelines reduced the number of low-abundant, potentially spurious amplicon sequence variants (ASVs) in the data sets, which led to lower dissimilarity between replicates. Analysis with a combination of Hill-based indices and a null model allowed us to show that different ecological mechanisms acted on different fractions of the microbial communities in the experimental systems.

**Conclusions:**

Hill-based indices provide a rational framework for analysis of dissimilarity between microbial community samples. In combination with a null model, the effects of deterministic and stochastic community assembly factors on taxa of different relative abundances can be systematically investigated. Calculations of Hill-based dissimilarity indices in combination with a null model can be done in qdiv, which is freely available as a Python package (https://github.com/omvatten/qdiv). In qdiv, a consensus table can also be determined from several count tables generated with different bioinformatic pipelines.

Video Abstract

## Background

Microbial communities drive global cycles of elements and play important roles for human health, food production, and environmental engineering services such as wastewater treatment. On Earth, there may be as many as 10^12^ different microbial species [1], and understanding how communities assemble, develop, and function is a formidable task. During the last decades, significant progress in DNA sequencing technology has provided a wealth of information about the diversity of microbial communities in both natural and engineered environments. Polymerase chain reaction (PCR) amplification of parts of the 16S rRNA gene followed by high-throughput sequencing using platforms such as 454 pyrosequencing, Illumina, Ion Torrent PGM, and PacBio has made it possible to probe millions of sequences in samples. For example, the Illumina MiSeq platform and dual-indexing of PCR primers allow over 100 samples to be sequenced in parallel at a depth exceeding 10,000 reads per sample [2, 3]. In addition to the rRNA gene, PCR targeting functional genes, such as the *amoA* in ammonia-oxidizing bacteria, can be used to study specific functional groups [4].

Interpretation of results from high-throughput amplicon sequencing experiments is, however, challenging. Varying copy numbers of the target gene, sampling, DNA extraction, PCR amplification, and sequencing can all lead to biases, which distort the relative proportions of taxa in a sample [5–7]. For example, Gonzalez et al. [8] showed that taxa with low abundance are typically underrepresented in PCR-based assays. PCR and sequencing also produce error-containing sequences [9]. Several computational pipelines can be used to differentiate between correct and erroneous sequence reads. After quality filtering, the reads are typically clustered into operational taxonomic units (OTUs), which are formed by grouping sequences that are similar. A similarity threshold of 97% has commonly been used. Recently, instead of OTU-clustering, alternative approaches have been developed that denoise the reads and derive exact biological sequences [10–12]. The denoiser algorithms use different methods to differentiate between true amplicon sequence variants (ASVs) and errors. The generated ASVs can differ from each other by as little as one nucleotide, which makes it possible to investigate microbial diversity at higher resolution (e.g., [13]). Another advantage is that the ASVs represent true biological entities and can be compared to results from other sequencing runs. In OTU clustering, the centroid sequences which represent the OTUs, as well as the classification of a read to an OTU, depend on all the other sequences in the run [14]. Thus, OTU sequences do not have a meaning outside of the specific context in which they are generated [15].

Once OTUs or ASVs have been determined, it is often of interest to study compositional differences between microbial communities in samples collected from different locations or time points (beta diversity). Indices describing the similarity or difference between sampled communities using a single number are commonly used. Many dissimilarity indices are available [16, 17]. Some, such as the Jaccard and Sørensen indices, are incidence-based, which means they do not consider differences in relative abundance between OTUs/ASVs. Other indices take the relative abundance into account. In microbial community assays, it is difficult to know how much weight should be put on the relative abundance of individual OTUs/ASVs. On the one hand, we know that the read abundance and the true relative abundance of microorganisms do not always correlate in PCR-based assays [18]. Rare OTUs/ASVs often are underrepresented [8] but can play important roles for community function [19]. It may therefore be tempting to use indices that weigh detected OTUs/ASVs equally. On the other hand, we know that PCR and sequencing cause errors, which may remain in the dataset after bioinformatics processing [9, 20]. Microbial communities typically also contain a long tail of extremely low-abundant taxa, and random sampling affects the observed dissimilarity [5]. This view would favor the use of an index giving higher weight to abundant OTUs/ASVs, and indeed, the Bray-Curtis index, which takes relative abundance into account, is probably the most commonly used taxonomic dissimilarity index in microbial ecology (equations for the Jaccard and Bray-Curtis indices are shown in Text S1.1, Additional file 1). The Bray-Curtis index is very sensitive to differences in relative abundance for the most abundant OTUs/ASVs, and a way to amplify the importance of differences for low-abundant OTUs/ASVs is to log-transform the count data before calculating the index [21]. However, a systematic approach for evaluating how relative abundance information affect observed dissimilarity is lacking for the indices described above.

There are, however, other indices that deserve more attention. Hill numbers are a set of diversity indices for which the weight given to the relative abundance of an OTU/ASV can be varied [22]. Hill numbers, which are also called effective numbers, were originally presented as measures of alpha diversity, i.e., OTU/ASV diversity within a community [23]. Equations 1a and 1b show how Hill numbers are calculated. The diversity order (*q*) determines the weight given to the relative abundance of an OTU/ASV in a community. For example, if *q* is 0, the relative abundance is not considered; if *q* is 1, the OTUs/ASVs are weighted exactly according to their relative abundance; and if *q* is higher than 1, more weight is given to OTUs/ASVs having high relative abundance. For a community with *S* OTUs/ASVs, all having the same relative abundances (i.e., 1/*S*), the Hill number is equal to *S* for all diversity orders.

$$ {}^qD={\left({\sum}_{i=1}^S{p}_i^q\right)}^{1/\left(1-q\right)}\kern5.2em \left(\mathrm{Eq}.1\mathrm{a},\kern0.5em \mathrm{if}\;\mathrm{q}\ne 1\right) $$

$$ {}^1D=\mathit{\exp}\left(-{\sum}_{i=1}^S\left({p}_i\cdot \mathit{\ln}\left({p}_i\right)\right)\right)\kern0.5em \left(\mathrm{Eq}.1\mathrm{b},\kern0.5em \mathrm{if}\;\mathrm{q}=1\right) $$

*D* is the Hill number, *q* is the diversity order, *S* is the total number of OTUs/ASVs, and *p*_*i*_ is the relative abundance of the *i*th OTU/ASV in the community.

For two or more communities, Hill numbers can be decomposed into alpha (*α*), gamma (*γ*), and beta (*β*) components [24]. ^*q*^*D*_*α*_ is the effective number of OTUs/ASVs per community (for a more detailed definition, see Text S1.2 in Additional file 1), ^*q*^*D*_*γ*_ is the Hill number for the combined communities (i.e., the regional or pooled community), and ^*q*^*D*_*β*_ is the ratio between the two (Eq. 2).

2 $$ {{}^qD}_{\beta }=\frac{{{}^qD}_{\gamma }}{{{}^qD}_{\alpha }} $$

The parameter ^*q*^*D*_*β*_ represents the effective number of distinct communities. It ranges from one to the number of communities being compared (*N*). If ^*q*^*D*_*β*_ = 1, the compared communities are identical to each other. If ^*q*^*D*_*β*_ = *N*, the compared communities are completely distinct and do not share any OTUs/ASVs with each other. ^*q*^*D*_*β*_ can be transformed to an overlap or dissimilarity index constrained between 0 and 1 (dissimilarity = 1 − overlap) [25]. There are several ways of doing this transformation [26]. Chao and Chiu [27] describe two classes of overlap indices. The *local* overlap indices measure the effective average proportion of OTUs/ASVs in a community shared with the other compared communities. The *regional* overlap indices measure the effective proportion of OTUs/ASVs in the pooled community that are shared between all compared communities. At a diversity order of 0, which means only the presence/absence of OTUs/ASVs is considered, the local index equals the Sørensen index and the regional index equals the Jaccard index. Equations 3a and 3b show the transformation of ^*q*^*D*_*β*_ into the class of local dissimilarity indices (^*q*^*d*). Thus, ^*q*^*d* quantifies the effective average proportion of OTUs/ASVs in a community *not shared* with the other compared communities. Throughout the article, we use this local class of indices when we refer to Hill-based dissimilarity. Further details about the calculations and equations for the class of regional indices can be found in Text S1.2, Additional file 1.

$$ {}^qd=\frac{{\left({{}^qD}_{\beta}\right)}^{\left(1-q\right)}-1}{N^{\left(1-q\right)}-1}\kern0.5em \left(\mathrm{Eq}.\kern0.5em 3\mathrm{a},\kern0.5em \mathrm{if}\kern0.5em \mathrm{q}\ne 1\right) $$

$$ {}^1d=\frac{\mathit{\ln}\left({{}^qD}_{\beta}\right)}{\mathit{\ln}(N)}\kern0.5em \left(\mathrm{Eq}.3\mathrm{b},\mathrm{if}\kern0.5em q=1\right) $$

^*q*^*d* is the local dissimilarity index of diversity order *q* and *N* is the number of communities being compared.

The use of Hill numbers is more common in the macroecological literature, both as measures of alpha diversity and for partitioning of diversity [28]. For microbial community studies using high-throughput amplicon sequencing, Hill numbers have also been recommended as measures of alpha diversity [29–31]. However, Hill-based indices are rarely used to quantify beta diversity. In two recent studies, we used Hill-based dissimilarity indices of specific diversity orders to quantify differences between microbial communities, giving different weight to the relative abundance of OTUs/ASVs [32, 33]. In this paper, we will show that examining dissimilarity (^*q*^*d*) for a continuum of diversity orders is a rational approach to illustrate how OTUs/ASVs with different relative abundances contribute to the dissimilarity between communities.

A difficulty with analyzing beta diversity, irrespective of the chosen index, is the interpretation of the results. We might be interested in determining if deterministic factors select for the same or different OTUs/ASVs in two sampled habitats or if the distribution of OTUs/ASVs between the habitats is governed by stochastic factors. The dissimilarity value alone tells us nothing about this. For example, if two habitats have different areas for microbial growth, the habitat with the larger area will likely have higher richness (number of detected OTUs/ASVs) because of the taxa-area relationship [34]. Since alpha and beta diversity are not independent (Eq. 2), the richness difference will cause a high observed dissimilarity even if the two habitats select for the same OTUs/ASVs [35, 36]. Null models are useful in the interpretation of dissimilarity values and allow us to differentiate between different community assembly mechanisms [36, 37]. A null model introduced by Raup and Crick [38] and developed by Chase et al. [36] controls for richness differences between samples. Samples with pre-defined numbers of OTUs/ASVs are randomly assembled from a regional pool. The definition of the regional pool and the randomization scheme will affect the outcome of a null model analysis [39, 40]. The regional pool could consist of all OTUs/ASVs detected in the samples being compared and could also include other OTUs/ASVs that could possibly colonize the studied habitat. The randomization scheme could, e.g., be based on the frequency of samples in which a certain OTU/ASV is found [41] or the total abundance of reads associated with the OTU/ASV in the regional pool. The random assembly process is repeated many times, and a null distribution for the dissimilarity between the two samples is generated. This null distribution is then compared to the observed dissimilarity. If the values are similar, the observed dissimilarity can be explained by stochastic factors. If the observed dissimilarity is higher or lower than the null expectation, there are likely deterministic factors that favor different or similar taxa in the two habitats [37]. The Raup-Crick model was originally developed for incidence-based data [36, 38] and was recently extended to also function with the Bray-Curtis index [41]. In this paper, we further extend the Raup-Crick null model to function with the whole continuum of Hill-based dissimilarity indices (^*q*^*d*) (Text S1.3, Additional file 1). The index, here denoted as the Raup-Crick index for diversity order *q* (^*q*^RC), is calculated using Eq. 4.

4 $$ {}^q\mathrm{R}\mathrm{C}=\frac{N_{\left[{{}^qd}_{\mathrm{exp}}<{{}^qd}_{\mathrm{obs}}\right]}+0.5\cdot {N}_{\left[{{}^qd}_{\mathrm{exp}}={{}^qd}_{\mathrm{obs}}\right]}}{N_{\mathrm{TOT}}} $$

*N*_*[qdexp<qdobs]*_ is the number of randomizations in which the dissimilarity between the randomly assembled samples is less than between the observed samples, *N*_*[qdexp=qdobs]*_ is the number of randomizations in which the dissimilarities are equal, and *N*_TOT_ is the total number of randomizations.

The goal of this study is to show how the choice of dissimilarity index impacts the results from high-throughput amplicon sequencing experiments. We examine sequencing data from a new experiment with aerobic granular sludge (AGS) reactors, and we re-analyze a previously published data set [32] from a study with microbial fuel cells (MFCs). To reduce the effects of bioinformatics choices on the sequencing results, we examine count tables generated with several bioinformatics pipelines and use a consensus approach to infer a count table that only includes ASVs detected by two different denoiser pipelines. In the AGS experiment, we test the hypothesis that two bioreactors started from the same inoculum and operated under identical conditions for 150 days exhibit the same change in microbial community composition compared to the inoculum. In the MFC experiment, we test the hypothesis that microbial communities growing in different habitats within a glucose-fed MFC are more similar than microbial communities growing in different habitats within an acetate-fed MFC. We show that the conclusions from an experiment may differ depending on the chosen dissimilarity index. We propose that a solution to this problem is to analyze community dissimilarity for a span of diversity orders using Hill-based indices, and we demonstrate that for the whole range of dissimilarity indices, null models can be used to disentangle community assembly mechanisms. Finally, we introduce a free software and Python package, qdiv, which enables rapid and simple calculations of the indices and includes an algorithm for the generation of consensus count tables. Our study focuses on taxonomic dissimilarity indices. The presented methods could, however, be extended to indices taking phylogenetic relationships into account.

## Results

### Behavior of Hill-based dissimilarity indices and the ^*q*^RC null model

**Fig. 1:**
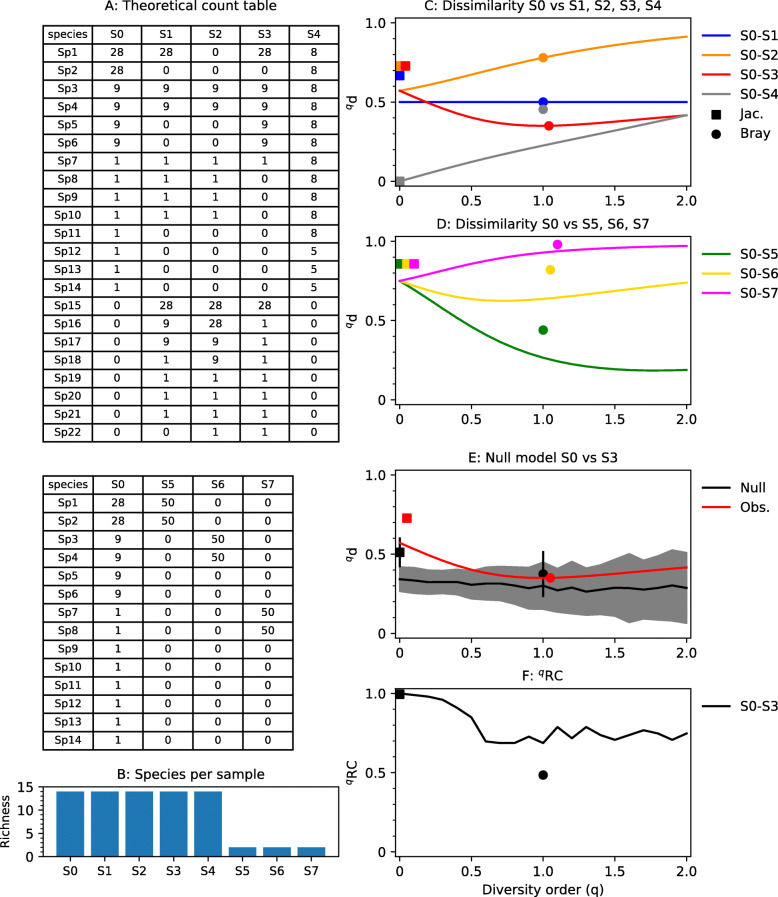
Behavior of dissimilarity indices with a theoretical data set. **a** Theoretical count table and **b** richness of each sample. **c** Behavior of dissimilarity indices for samples with equal species abundance distribution, sharing exactly half of the abundant, intermediate, and rare species (S0–S1); sharing no abundant but half of the rare and intermediate species (S0–S2); or sharing all the intermediate species but only half of the rare and abundant (S0–S3). S0–S4 share all species but have different species abundance distributions. **d** Behavior of dissimilarity indices for samples having different richness (14 in S0 and 2 in S5–S7). In S0–S5, the shared species are the same as the most abundant in S0; in S0–S6, the shared species are those of intermediate abundance in S0; and in S0–S7, the shared species are rare in S0. **e**, **f** Null model analysis comparing observed dissimilarity to the null expectation for samples S0–S3. The black line and shaded region in **e** show the average and standard deviation for the null expectation based on 99 randomizations. Observed dissimilarity and the null expectation (**e**), and ^q^RC values (**f**) for the Jaccard (squares) and Bray-Curtis (circles) indices are also shown

Count tables from microbial community surveys typically consist of a few highly abundant OTUs/ASVs and many low-abundant ones. Using a highly simplified count table (Fig. 1a, b), we demonstrate how the Hill-based dissimilarity indices behave in comparison to the Jaccard and Bray-Curtis indices, which are more commonly used in microbial community studies. Hill-based dissimilarity (^*q*^*d*) is shown as functions of the diversity order, *q* (Fig. 1c, d). Since the Jaccard index is identical to the regional Hill-based dissimilarity index of diversity order 0 (Text S1.2, Additional file 1), it is plotted at *q* equals 0. The Bray-Curtis index is plotted at *q* equals 1. Bray-Curtis and Hill-based dissimilarity indices are usually not comparable. However, in the special case when two samples have the same species abundance distribution and a species detected in both samples have the exact same relative abundance in both samples, the Bray-Curtis dissimilarity is identical to ^1^*d* (for proof, see Text S1.4 in Additional file 1).

First, let us consider the situation when samples have equal richness, i.e., the same numbers of detected species (Fig. 1c). Four samples (S0, S1, S2, S3) each have 2 abundant, 4 intermediate, and 8 rare species. Samples S0 and S1 share 1 abundant, 2 intermediate, and 4 rare species. As expected, the Hill-based dissimilarity (^*q*^*d*) between S0 and S1 is 0.5 for all values of *q*. Sample S0 and S2 share half of the rare and intermediate species, but none of the abundant species and consequently ^*q*^*d* goes towards 1 as *q* increases. Samples S0 and S3 share all intermediate species, but only 1 of the abundant and 1 of the rare, and consequently, we see a valley in the ^*q*^*d* vs *q* curve. In these special cases, both samples have the same species abundance distribution and a species detected in both samples have the exact same relative abundance in both samples. Consequently, the Bray-Curtis dissimilarity is identical to ^1^*d*. Sample S4, however, has the same richness as S0 but a different species abundance distribution, and the Bray-Curtis index is different from ^1^*d*.

Second, let us consider the situation when samples have unequal richness (Fig. 1d). Samples S5–S7 have only two species each. In S5, those two species are the same as the most abundant ones in sample S0, and consequently, ^*q*^*d* decreases with increasing q. In S6, the two species are the same as two intermediates in S0 and we can see a valley in the curve. In S7, the two species are the same as two rare ones in S0 and the dissimilarity increases with *q*. The Bray-Curtis index shows a different behavior. For S0–S5, Bray-Curtis is equivalent to Hill-based dissimilarity with a low diversity order (*q*) of 0.52, and for S0–S6 and S0–S7, it is equivalent to diversity orders (*q*) much higher than 2.

Using the ^*q*^RC null model, we can compare the observed dissimilarity between two samples to the expected dissimilarity if the two sampled communities had been randomly assembled from a regional species pool. The ^*q*^RC values, as calculated in Eq. 4, are constrained between 0 and 1. A value close to 0 means lower dissimilarity than the null expectation, and a value close to 1 means higher dissimilarity than the null expectation. In Fig. 1e, f, the sample pair S0–S3 is used as an example. For values of *q* close to 0, the observed dissimilarity is higher than the null expectation and consequently ^0^RC is 1. For higher values of *q*, the observed dissimilarity is close to the null expectation and consequently the ^q^RC values are intermediate, i.e., neither close to 0 or 1 (Fig. 1f). For this theoretical example, it means that if we weigh species according to their relative abundance (*q* ≈ 1), the observed dissimilarity could be explained by random assembly of the two communities from the regional species pool, but if we give equal weight to all species (*q* ≈ 0), the observed dissimilarity is higher than we can expect from a random assembly process.

### Inferring consensus count tables from the experimental data

The number of low-abundant OTUs/ASVs detected when microbial communities are analyzed using high-throughput amplicon sequencing can be highly dependent on bioinformatics pipeline [42]. Here, we compare results using several pipelines operated with different settings and infer a consensus table based on the output from two denoiser pipelines. Samples collected from two experiments (AGS and MFC) were sequenced in two separate sequencing runs. The sequences were processed using DADA2 version 1.10 [43], Deblur version 1.04 [44], USEARCH version 10 [45], and Mothur version 1.41 [46] with various settings, resulting in 11 count tables for each experiment. In USEARCH, we used both UNOISE to determine ASVs and UPARSE to cluster OTUs (see Text S2.1 in Additional file 2). There were large differences in the number of detected OTUs/ASVs by different pipelines. This was mostly caused by large numbers of low-abundant, potentially spurious OTUs/ASVs appearing when the pipelines were run with relaxed quality filtering thresholds. Despite the large richness differences, count tables generated with different pipelines generally had similar abundance-based diversity values and evenness. They also showed similar beta diversity patterns and were able to distinguish between different sample categories in the data sets (see Text S2.3-4 in Additional file 2).

**Fig. 2:**
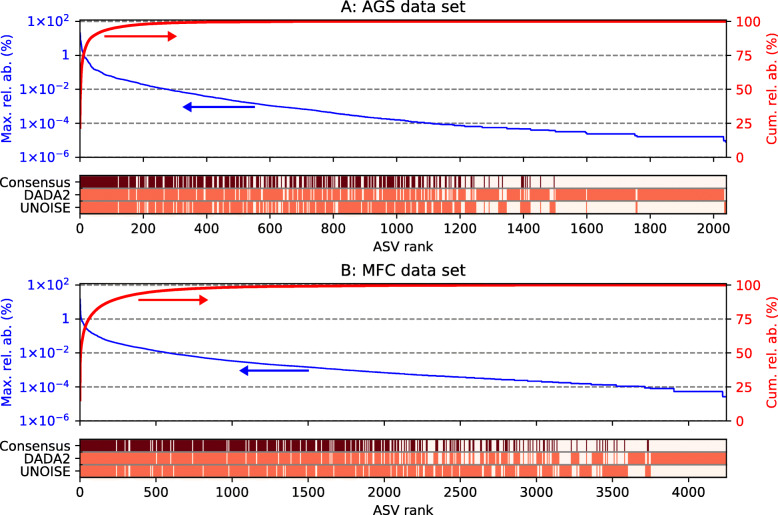
Relative abundance (%) of ASVs retained in the consensus tables for the AGS (**a**) and MFC (**b**) data sets. Each ASV in the two input tables, arranged from highest to lowest relative abundance, is shown on the *x*-axis. The blue lines show the maximum relative abundances of the ASVs in the DADA2 and UNOISE count tables, and the red lines show the cumulative relative abundances. The heatmaps show whether the ASVs were detected in the DADA2 and UNOISE count tables (light red). If it was detected in both, it was also retained in the consensus table, which is indicated by dark red color

Denoiser pipelines generate exact ASVs, which represent true biological entities. Thus, an ASV found with one denoiser pipeline should also be found with another. To filter out potentially spurious ASVs, information from several pipelines can be combined in a consensus table. A function for generating a consensus table from an unlimited number of count tables was implemented in qdiv. The consensus function identifies ASVs that are detected in all compared count tables. For each count table, the fraction of the reads associated with the set of shared ASVs is calculated. The count table with the highest fraction is retained, all ASVs not belonging to the shared set are discarded, and the retained count table with the remaining shared ASVs is returned as the consensus table (for a more detailed description, see Text S2.2 in Additional file 2). In this study, we inferred a consensus table based on two count tables generated with DADA2 and UNOISE. For the AGS data set, the DADA2 and UNOISE count tables had 1768 and 1192 ASVs, respectively. The consensus function identified 919 shared ASVs. The UNOISE count table had 99.7% of its read counts mapped to these shared ASVs and was retained as the consensus table after being subsetted to the shared ASVs. For the MFC data set, the DADA2 and UNOISE count tables had 3355 and 3152 ASVs, respectively. The consensus table was based on the UNOISE table, which had 99.4% of its reads mapped to the 2258 shared ASVs. The relative abundances of the ASVs detected by the count tables are shown in Fig. 2. The ASVs that are not retained in the consensus table have low relative abundance spanning from 8 × 10^−6^ to 0.05% in the AGS data set and 3 × 10^−6^ to 0.8% in the MFC data set. Before analysis of dissimilarity, the count tables were rarefied to the number of reads in the smallest sample. This was 278,758 reads/sample in the AGS data set and 33,171 reads/sample in the MFC data set. Further details about the count tables are shown in Fig. S2.1-10 in Additional file 2.

The consensus count tables were used to evaluate dissimilarity between replicate samples and test hypotheses on the experimental data from the AGS and MFC systems.

### The observed dissimilarity between replicates is affected by the choice of dissimilarity index

Both the AGS and MFC samples contained microbial community replicates, which means that DNA was extracted in parallel from six aliquots of biomass collected from the same microbial community (e.g., the same AGS reactor or the same MFC biofilm). The MFC samples also contained one set of technical replicates, which in this study means that the same DNA extract was processed in six separate PCR reactions followed by sequencing of the six separate PCR products.

**Fig. 3:**
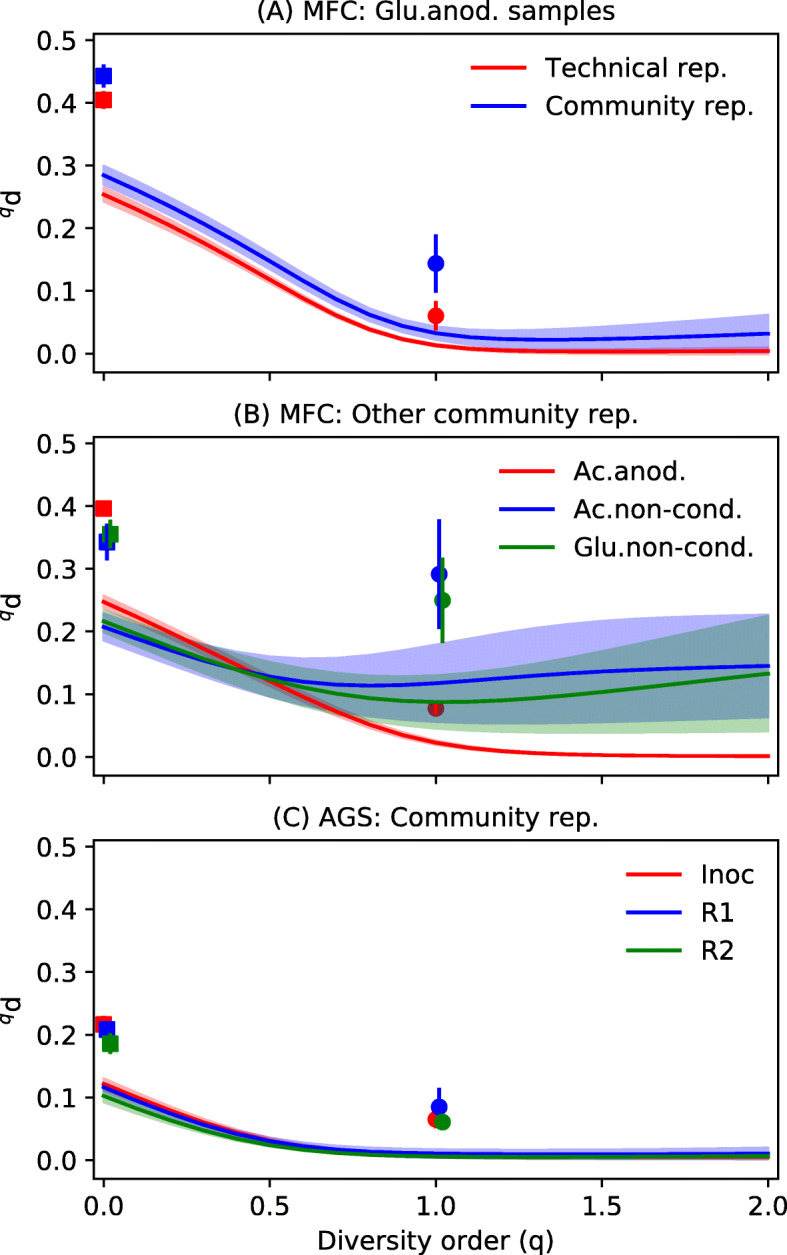
Dissimilarities between replicates (*n* = 6). **a** A comparison between the community and technical replicates for samples from the MFC experiment. **b** Other community replicates from the MFC experiment and **c** community replicates from the AGS experiment. Hill-based dissimilarity values (^*q*^*d*) are shown as lines. Jaccard and Bray-Curtis dissimilarities are shown as squares and circles, respectively. Shaded regions and error bars are standard deviations of pairwise dissimilarities (*n* = 15). The MFC data set had four categories of samples: acetate-fed biofilms growing on anodes (Ac.anod.), acetate-fed biofilms growing on non-conductive surfaces (Ac.non-cond.), glucose-fed biofilms growing on anodes (Glu.anod.), and glucose-fed biofilms growing on non-conductive surfaces (Glu.non-cond.). The AGS data set had three sample categories: the inoculum (Inoc), reactor 1 (R1), and reactor 2 (R2). The technical replicates were taken from a Glu.anod. sample

The diversity order (*q*) of the dissimilarity index had a strong effect on the dissimilarity between replicates. The highest dissimilarity was observed for incidence-based indices (^0^*d* and Jaccard), and the dissimilarity typically decreased with increasing diversity order (Fig. 3). Overall, the technical replicates had lower dissimilarity than the community replicates for diversity orders from 0 to 2 (*p* < 0.05, *n* = 15, Welch’s anova). The consensus table had lower dissimilarity between replicates than the two count tables used to generate the consensus table at low diversity orders (*q* < 1) for all seven sets of community replicates as well as for the technical replicates (see Fig. S2.12 in Additional file 2).

### Random sampling affects the observed dissimilarity between replicates

**Fig. 4:**
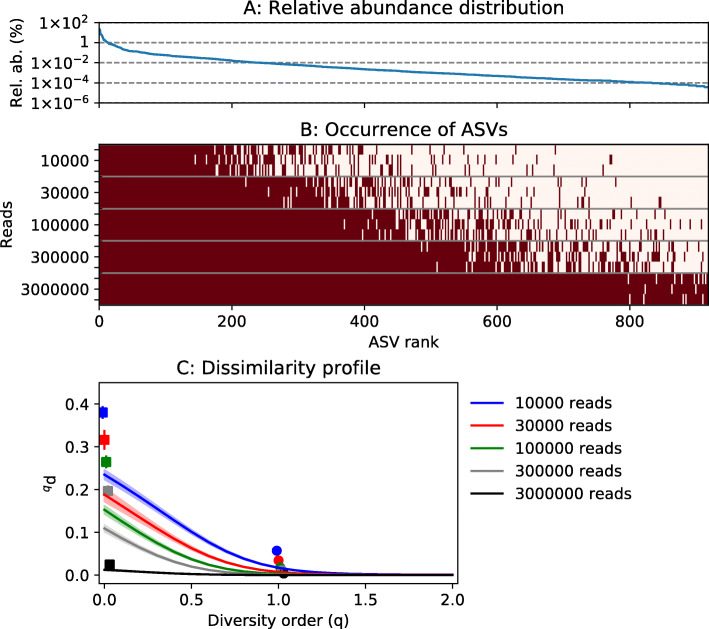
Simulation of the effect of sequencing depth on dissimilarity between replicates. **a** Relative abundance distribution for the microbial community being sampled. **b** ASVs detected in samples having different sequencing depths. Dark red color indicates that the ASV was detected. Three samples are shown for each sequencing depth. **c** Average pairwise dissimilarities between replicate samples at each sequencing depth. The shaded regions show the standard deviations (*n* = 15). Jaccard- and Bray-Curtis dissimilarities are shown as squares and circles, respectively

The high dissimilarity between replicates for low diversity orders could be the result of undersampling [47]. To examine this effect, we used a simulation. The AGS data set served as a hypothetical case. Figure 4a shows the relative abundance distribution of the 919 ASVs found in the AGS consensus table. Let us assume this represents the true relative abundances of all taxa present in the investigated microbial community. Five sets of samples with sequencing depths ranging from 10,000 to 3 million reads per sample were obtained from the community. The samples were generated by random sampling with replacement from the relative abundance distribution. Increasing sequencing depth led to increasing number of detected ASVs (Fig. 4b). The average pairwise dissimilarity between six replicate samples is shown in Fig. 4c. The curves have the same shape as the experimentally observed dissimilarities in Fig. 3. A sequencing depth of 300,000, which is similar to the actual sequencing depth for the AGS data set (278,758 reads/sample), generated approximately the same dissimilarity profile as the real data (see Figs. 3c and 4c). The detection of the ASVs increased, and the dissimilarity between replicates decreased with increasing sequencing depth (Fig. S2.13, Additional file 2). At a sequencing depth of 3 million reads, 98.5 ± 0.4% of the ASVs were detected.

### Effect of the choice of diversity index on observed differences between sample categories

The ability of different dissimilarity indices to distinguish between sample categories in the experimental data was also tested. The AGS data set was more challenging than the MFC data set because most taxa were shared between different samples. Therefore, the AGS consensus table with the three sample categories, the inoculum, reactor 1 (R1), and reactor 2 (R2), was used in the analysis. The *F*-statistic is the ratio of between-group variability and within-group variability. Dissimilarity matrices resulting in the calculation of a high *F*-statistic are thus better at resolving differences between sample categories. Dissimilarity matrices generated with the ^1^*d* and ^2^*d* indices resulted in *F*-statistics of 2492 and 2969, respectively. The Bray-Curtis index resulted in an *F*-statistic of 153. The incidence-based ^0^*d* and Jaccard indices resulted in values of 20 and 15, respectively. High dissimilarity between replicates, which was observed for the incidence-based indices (Fig. 3), would result in lower *F*-statistic. Despite large differences in the *F*-statistic, statistically significant separation between the three sample categories was found with all dissimilarity indices (permanova, *p* = 0.001, 999 permutations) (see also Text S2.4 in Additional file 2). A PCoA showing separation between the sample categories using the ^0^*d* index is shown in Fig. S2.11 (Additional file 2).

### The choice of dissimilarity index influence hypothesis testing

#### AGS experiment

**Fig. 5:**
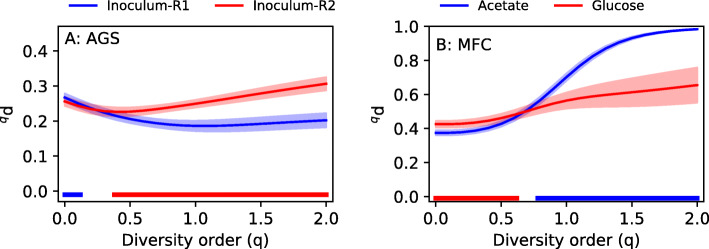
**a** Average pairwise dissimilarity between the inoculum and R1, and the inoculum and R2 for the AGS data set. **b** Average pairwise dissimilarity between the electroactive biofilm growing on the anode and the biofilm growing on the non-conductive separator in the acetate-fed and glucose-fed MFCs. Shaded regions show standard deviations. The horizontal bars near the *x*-axis indicate significant difference in dissimilarity (Welch’s anova, *p* < 0.05, *n* = 36). The color of the bar shows which pair has the highest dissimilarity

In the AGS experiment, we hypothesized that R1 and R2 diverged from the inoculum to the same extent after 150 days of operation since they were operated under identical condition and had similar performance. Thus, the dissimilarity between the inoculum and R1 should be the same as between the inoculum and R2. The results are shown in Fig. 5a. For high diversity orders (*q* ≥ 0.4), the dissimilarity between the inoculum and R2 is larger than between the inoculum and R1, and for low diversity order (*q* ≤ 0.1), higher dissimilarity is observed between the inoculum and R1 (*p* < 0.05, Welch’s anova). However, it should be noted that the magnitude of the difference is small at low diversity order.

#### MFC experiment

In the MFC experiment, we compared microbial communities of electroactive biofilms growing on anodes with biofilms growing on non-conductive porous separators. We hypothesized that biofilms growing on conductive and non-conductive surfaces would be more dissimilar to each other in the acetate-fed MFC than in the glucose-fed MFC. Glucose is a fermentable substrate, and fermentative microorganisms should be able to grow anywhere within the MFCs, leading to a more homogenous microbial community structure. Acetate, on the other hand, is non-fermentable, and the microbial communities in an acetate-fed MFC are therefore dependent on electron acceptor availability. On the anode surface, the anode serves as an electron acceptor while in other locations within the MFCs, the microorganisms must use soluble compounds such as oxygen diffusing in through the gas-diffusion cathode. Microbial communities in different locations of the acetate-fed MFCs should therefore have different metabolisms, which likely leads to higher dissimilarity than between communities within the glucose-fed MFCs which, at least partly, could have the same metabolism, namely fermentation [32]. For high diversity orders (*q* ≥ 0.8), there was higher dissimilarity in the acetate-fed MFC than in the glucose-fed MFC. For low diversity orders (*q* ≤ 0.6), the glucose-fed MFC had higher dissimilarity (*p* < 0.05, Welch’s anova) (Fig. 5b).

#### Null model

**Fig. 6:**
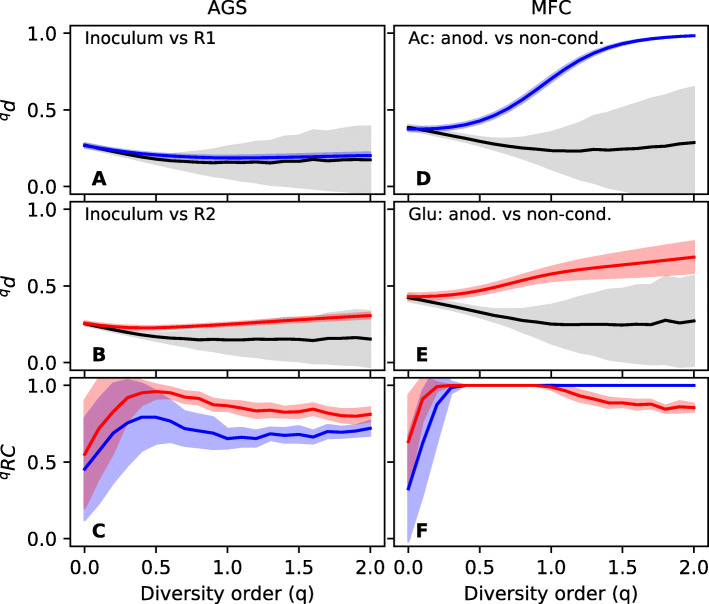
Null model simulation (199 randomizations). **a**–**c** Results for the AGS data set. **d**–**f** Results for the MFC data set. **a** Dissimilarity between the inoculum and R1 (blue) in comparison to the null distribution (black). **b** Dissimilarity between the inoculum and R2 (red) in comparison to the null distribution (black). **c** ^*q*^RC values for the inoculum-R1 (blue) and inoculum-R2 (red) comparisons. **d** Dissimilarity between biofilms on anodes and non-conductive surfaces in the acetate-fed MFC (blue) in comparison to the null distribution (black). **e** Dissimilarity between biofilms on anodes and non-conductive surfaces in the glucose-fed MFC (red) in comparison to the null distribution (black). **f** ^*q*^RC values for the biofilm comparisons in the acetate-fed MFC (blue) and glucose-fed MFC (red). Shaded regions show standard deviations based on all pairwise comparisons (*n* = 36)

Null models were used to aid in the interpretation of dissimilarity values. The results from the AGS experiment are shown in Fig. 6**a**–**c**. The dissimilarity between the inoculum and R1 is not significantly different from the null distribution at any diversity order, and consequently, ^*q*^RC is close to 0.5. For the inoculum and R2, the observed dissimilarity is higher than between the inoculum and R1; however, the null expectation of random assembly could not be rejected at a significance level of 0.05.

For the MFC data set, the results from the null model analysis are shown in Fig. 6**d**–**f**. At a diversity order of 0, the observed dissimilarity is similar to the null expectation, and consequently, ^*q*^RC is close to 0.5. This indicates that if we only care about presence/absence of ASVs, there is a random distribution between the two biofilm communities. With increasing emphasis on relative abundance, the dissimilarity between biofilm types is higher than the null distribution. For the acetate-fed MFCs, the ^*q*^RC values are close to 1, which means significant compositional differences between the two communities. For the glucose-fed MFCs, the ^*q*^RC again drops to lower values at a diversity order above 1. This means that some of the most abundant ASVs are shared between biofilms growing on conductive and non-conductive surfaces. This indeed turned out to be the case with a *Trichococcus* sp. being highly abundant in both biofilm communities, likely carrying out fermentation in both places [32].

## Discussion

### A consensus count table removes many low-abundant ASVs but retains most of the reads

Previous studies comparing bioinformatics pipelines for high-throughput sequencing of marker genes have found large differences in alpha diversity estimates [42, 48–51]. We also observed that both the pipeline and the input parameter values chosen by the user affected the number of inferred OTUs/ASVs as well as the number of reads mapped to these (see Fig. S2.1-2 in Additional file 2). With real samples of unknown composition, it is difficult to choose which pipeline and which settings to use for the analysis. A way to approach the problem of inflated OTU/ASV counts is to infer a consensus table based on ASVs detected using several different pipelines. We have implemented an algorithm for doing this in qdiv. Running the algorithm with DADA2 and UNOISE count tables as input resulted in dramatic drops in the ASV counts in the consensus tables; however, most of the reads (99.4–99.7%) were associated with the consensus ASVs.

### Dissimilarity between replicates depends on the diversity order and can be explained by random sampling effects

High dissimilarity between replicates can make it difficult to use marker-gene amplicon sequencing to distinguish categories of samples. For example, Bautista-de los Santos et al. [52] studied microbial communities in drinking water using the Jaccard and Bray-Curtis indices on an OTU table generated with Mothur. Fewer significant differences between sample categories were observed with the Jaccard index because of high dissimilarity between replicate samples [52]. We also observed much lower *F*-statistics for the AGS data set with incidence-based dissimilarity indices, which was caused by higher dissimilarity between community replicates in relation to dissimilarity between sample categories.

Dissimilarity between replicates can be caused by many factors associated with sampling, DNA extraction, PCR, sequencing, and data processing [53]. The comparison between community and technical replicates in Fig. 3a suggested that only a relatively small fraction was associated with sampling and DNA extraction for the case of an MFC biofilm sampled from an anode. The dissimilarity of replicates was the highest for incidence-based indices and low diversity order (*q* < 1), which means that low-abundant ASVs had a strong influence on the observed dissimilarity. The species-abundance distribution of microbial communities can contain a long tail of low-abundant taxa of which only some may be detected in the analyzed samples. This random sampling effect [5, 47], as well as the generation of erroneous OTUs/ASVs during PCR, sequencing, and data processing, causes dissimilarity between replicates. The random sampling effect was shown using a simulation in Fig. 4, where the simulated dissimilarity between replicates corresponded very well with the experimentally observed dissimilarity at a sequencing depth of approximately 300,000 reads/sample.

Previously, Haegeman et al. [31] showed the difficulty of estimating alpha diversity at low diversity orders (*q* < 1) because even in deeply sequenced samples, we lack information about the tail of low-abundant OTUs/ASVs. In the simulation in Fig. 4, the true dissimilarity was 0 since all samples were collected from the same hypothetical community. However, the simulated dissimilarity for low diversity orders (*q* < 1) was much higher than 0, although it decreased as sample size increased.

Figures 3, 4, and 5 show dissimilarity as a function of diversity order. The mean and standard deviation of several pairwise comparisons of samples from the compared communities are shown in each figure. Although we know that the calculated dissimilarities at low diversity order are likely incorrect, the standard deviations (shaded regions) are generally very small. This means that for a given sample size (sequencing depth), the calculated dissimilarity is reproducible. It does not mean that the calculated dissimilarity is a good estimate of the true dissimilarity between the microbial communities from which the samples were taken. For example, Fig. 4 shows the mean and standard deviation of 15 pairwise dissimilarity values between six simulated samples. The standard deviation of the simulated dissimilarity is very small, but the mean is far from the true value. For a sample size of 300,000 reads, ^0^*d* was 0.11 ± 0.01. However, in this case, we know that the true dissimilarity was 0.

The dissimilarity between replicates decreased with increasing diversity order until *q* was approximately one (Fig. 3). For some samples, most notably the biofilm samples from non-conductive surfaces in the MFC experiment, the dissimilarity between replicates then increased at higher diversity order and for the Bray-Curtis index (Fig. 3b). At low diversity order (*q* < 1), the dissimilarity between replicates could be lowered by generating a consensus table (Fig. S2.12, Additional file 2). The consensus table excludes many low-abundant and potentially spurious ASVs from the data sets. Since low-abundant OTUs/ASVs have a large impact on low diversity order dissimilarity indices, dropping some of them from the data set leads to reduced dissimilarity. At a high diversity order (e.g., *q* = 2), the calculated dissimilarity is highly dependent on the relative abundance of the most abundant OTUs/ASVs in each sample. Small differences in relative abundance values of those OTUs/ASVs are amplified, which leads to increasing dissimilarity. In the MFC sample, heterogeneity of the biofilms growing on the non-conductive surfaces may have caused the observed dissimilarity between community replicates at high diversity order. The ^1^*d* index, which weighs OTUs/ASVs exactly according to their relative abundance in the sample, seems to be a good compromise leading to low dissimilarity between replicates and hence better possibilities of detecting actual differences between samples collected from microbial communities exposed to different treatments.

### Hypotheses should be tested for a range of diversity orders to determine the effects of taxa with different relative abundances

Previous research has shown that Hill numbers are suitable for quantifying alpha diversity in samples obtained by high-throughput sequencing of marker-genes [29]. For example, Haegeman et al. [31] analyzed alpha diversity as a function of diversity order and concluded that Hill numbers with *q* > 1 give robust estimates of alpha diversity. In this study, we show that dissimilarity profiles, which show the dissimilarity between samples as a function of diversity order (Fig. 5), are highly informative also in the study of beta diversity. The use of a single dissimilarity index would have given misleading information for the data sets investigated in this study. In the AGS experiment, incidence-based indices showed that R1 and R2 were about equally dissimilar to the inoculum. However, at higher diversity order, there was a clear difference. In the MFC experiment, the incidence-based indices would have led us to conclude that the dissimilarity between biofilms on conductive and non-conductive surfaces in the acetate-fed MFCs was lower than in the glucose-fed MFCs, contrary to our hypothesis. However, when we plot dissimilarity as a function of *q*, we see that when we focus on the more abundant ASVs (*q* > 1), the bioanodes and biofilms in the glucose-fed MFCs are in fact less dissimilar, in line with our hypothesis.

Contrary to the commonly used Bray-Curtis index, the Hill-based dissimilarity indices have an intuitive interpretation. The ^*q*^*d* index quantifies the effective average proportion of OTUs/ASVs in one sample *not shared* with the other sample [54]. If two samples have S number of equally common OTUs/ASVs and C of them are shared, the dissimilarity value would be 1-C/S [25]. Thus, the number itself has a meaning. For example, ^0^*d* can be interpreted as the average proportion of all OTUs/ASVs-, ^1^d as the average proportion of “common” OTUs/ASVs-, and ^2^*d* as the average proportion of “abundant” OTUs/ASVs *not shared* between two samples.

The Hill-based dissimilarity indices can also be extended to take relationships between OTUs/ASVs into account [54]. Using either a phylogenetic tree or a matrix of pairwise distances as input, phylogenetic, or functional dissimilarity indices can be calculated [26, 55, 56]. As phylogenetically closely related taxa tend to have similar functional capabilities and habitat preferences [57], dissimilarity indices that take phylogenetic relatedness into account could, in comparison to taxonomic indices, provide more information about functional differences between microbial communities.

Null models help us to further interpret the meaning of the dissimilarity values. In the AGS experiment, reactors R1 and R2 were inoculated and then operated for 150 days. Ecological drift could have caused the microbial communities in the reactors to diverge from the inoculum. The null model tested if there was significant compositional turnover in R1 and R2 in comparison to the inoculum. However, the null expectation of random community assembly could not be rejected. Even for R2, which displayed the highest dissimilarity from the inoculum at *q*≥ 0.4, random assembly could explain the observed dissimilarity values. Thus, 150 days appears to be a too short time frame to see the effect of ecological drift in the studied system. The data set from the MFCs showed that for a diversity order of 0, the distribution of ASVs between the two types of biofilms was close to the null expectation. This is logical considering that the two biofilms were physically located close to each other and linked by dispersal. There is, thus, a high likelihood that the same ASVs can be detected in both locations, even if they do not grow in both locations. For higher diversity order (i.e., *q* = 1), we see a higher dissimilarity than the null expectation, suggesting that the common ASVs were different in the two locations. This could be explained by heterogeneous selection. The conductive anode surface selected for electroactive microorganisms whereas the non-conductive separator selected for oxygen scavengers. For even higher diversity order (*q* = 2), the dissimilarity between the two biofilms in the glucose-fed MFC again approaches the null expectation. This is logical considering that one of the most abundant taxa in the glucose-fed MFCs was a fermentative *Trichococcus* sp., which could grow in both locations [32].

## Conclusions

- Bioinformatics pipelines ran with different settings resulted in count tables having large differences in the number of OTUs/ASVs and total reads. A way to minimize the effect of low-abundant and possibly spurious OTUs/ASVs on the analysis is to generate a consensus table based on several other count tables generated using different denoising pipelines (e.g., UNOISE, DADA2, and Deblur).
- Conclusions drawn from experimental data can depend on the chosen dissimilarity index. To fully understand beta diversity patterns, Hill-based dissimilarity values should be calculated for several diversity orders (*q*). Dissimilarity profiles plotting ^*q*^*d* as a function of *q* are informative.
- Null models, which can be calculated based on all dissimilarity indices, help in the interpretation of dissimilarity values and give information about community assembly mechanisms.
- The Python package qdiv, freely available at https://github.com/omvatten/qdiv with documentation at https://qdiv.readthedocs.io/en/latest/, enables simple calculation of Hill-based dissimilarity indices and associated null models. It can also be used to calculate consensus count tables.

## Methods

### Experimental

Samples collected from two separate experiments were analyzed in this study. In the AGS experiment, granular sludge from a sequencing batch reactor was used to inoculate two new reactors (R1 and R2). Six samples were collected from the inoculum as well as from each of the two new reactors after 150 days of operation (Fig. S3.1, Additional file 3). The sets of six are called community replicates. Reactor R1 and R2 had similar performance over time with total organic carbon removal > 90% and total nitrogen removal of 35.2 ± 14.6% in R1 and 37.0 ± 12.7% in R2. They also had similar average granule size in the end of the experiment and followed the same trajectory in terms of suspended solids concentrations in the reactors.

In the MFC experiment, parallel MFCs were operated with either acetate or glucose as the sole electron donor [for details, see 32]. Samples were collected from the anode where a biofilm of electroactive microorganisms oxidized the electron donor and generated electrical current, and from a non-conductive porous separator where a biofilm oxidized or fermented the electron donor and scavenged oxygen (Fig. S3.2 Additional file 3). In one acetate- and one glucose-fed MFC, the biofilm samples were each cut into six pieces and DNA was extracted and processed separately from each piece. These samples are called community replicates. The DNA extracted from one of the anode-attached biofilm samples was also processed in six separate PCR reactions. These samples are called technical replicates.

DNA was extracted using the FastDNA Spin Kit for Soil (MP Biomedicals). PCR amplification of the V4 region of the 16S rRNA gene was carried out with the primer pair 515′F (GTGBCAGCMGCCGCGGTAA) and 806R (GGACTACHVGGGTWTCTAAT) [58, 59] and the dual indexing strategy by Kozich et al. [3]. High-throughput sequencing was carried out using the Illumina MiSeq platform and reagent kit V3 (2 × 300 bp paired-end sequencing). Further details are provided in Text S3.1 (Additional file 3). The samples from the AGS and MFC experiments were processed in two separate sequencing runs. The sequencing results were deposited in the European Nucleotide Archive with accession numbers PRJEB35721 (AGS data set) and PRJEB26776 (MFC data set). The specific run accession numbers for each pair of fastq files used in the study and the corresponding sample identities are shown in Tables S3.1-2 (Additional file 3).

### Bioinformatics

The sequence reads were processed using DADA2 version 1.10 [43], Deblur version 1.04 [44], USEARCH version 10 [45], and Mothur version 1.41 [46]. The pipelines offer the user various choices. For example, the stringency of the quality filtering method can typically be varied, and the reads can often be processed either separately sample-by-sample or in pooled mode. Analysis of pooled samples requires more computer memory. DADA2 and Deblur generate ASVs whereas Mothur generate OTUs. USEARCH can either generate ASVs using UNOISE [60] or OTUs using UPARSE [61]. Several count tables were generated using various input parameter settings in the pipelines (see Additional file 2). Details about the pipelines are provided at github.com/omvatten/amplicon_sequencing_pipelines. DADA2 and UNOISE count tables were used to generate consensus tables consisting of ASVs detected using both pipelines. This was done with a function implemented in qdiv.

### Software

A software, qdiv, allowing calculation of all the indices and null models mentioned above was developed in Python3 and is available as a Python package. It makes use of the following Python packages: pandas [62], numpy [63], matplotlib [64], and python-Levenshtein. The source code for qdiv is available at https://github.com/omvatten/qdiv. It is available via PyPI and the Anaconda cloud.

### Statistical analysis

To determine statistical significance of the association between different dissimilarity matrices, Mantel’s permutation test was used [65]. To compare the variability within sample categories to the variability between sample categories, permanova was used [66]. Both the Mantel test and permanova were implemented in qdiv. Welch’s anova was carried out using SciPy [67].

### Null model

In the AGS experiment, we defined all samples from the inoculum, R1, and R2 as the regional pool. In the MFC experiment, we were interested in the dissimilarity between the anode biofilm and biofilm growing on a non-conductive surface within the same MFC. Thus, we defined all samples collected from one specific MFCs as one regional pool. For randomization scheme, we used the frequency approach, which is the same as in Stegen et al. [41]. Briefly, the number of OTUs/ASVs and reads in a sample are recorded. The null version of the sample is generated by randomly picking the same number of OTUs/ASVs from the regional pool. The likelihood of being picked corresponds to the frequency of samples in which the OTU/ASV is found. The picked OTUs/ASVs are then populated with reads so that the total number of reads in the randomly assembled sample equals that of the real sample. The likelihood for a read of being picked is related to the total number of reads associated with the OTUs/ASVs in the regional pool.

It should be noted that the ^*q*^RC value defined in Eq. 4 is constrained between 0 and 1. If a range between − 1 and 1 is desired, e.g., as in Chase et al. [36], this can be accomplished by subtracting 0.5 from the ^*q*^RC value and multiplying by 2.

## Supplementary information

**Additional file 1.** Dissimilarity indices and null model

**Additional file 2.** Bioinformatics pipelines and consensus algorithm

**Additional file 3.** Experimental details

## Data Availability

Amplicon sequence data are deposited at the European Nucleotide Archive under accession numbers PRJEB35721 (AGS data set) and PRJEB26776 (MFC data set). Sample identities corresponding to the run accession numbers are provided in Table S3.1-2 (Additional file 3). Bioinformatics pipelines used to process the sequence data and generate count tables are available at https://github.com/omvatten/amplicon_sequencing_pipelines. Information about the execution of the pipelines is also provided in Text S2.1 (Additional file 2). The code for qdiv, which was the software developed in this project and used to analyze the count tables, is available at https://github.com/omvatten/qdiv.
