# Correction to: Hill-based dissimilarity indices and null models for analysis of microbial community assembly

**DOI:** 10.1186/s40168-020-00942-6

**Published:** 2020-10-28

**Authors:** Oskar Modin, Raquel Liébana, Soroush Saheb-Alam, Britt-Marie Wilén, Carolina Suarez, Malte Hermansson, Frank Persson

**Affiliations:** 1 Water Environment Technology, Architecture and Civil Engineering, Chalmers University of Technology, Gothenburg, Sweden; 2 Chemistry and Molecular Biology, University of Gothenburg, Gothenburg, Sweden

**Correction to: Microbiome 8, 132 (2020)**

**https://doi.org/10.1186/s40168-020-00909-7**

Following publication of the original article [1], it was identified that a statement was missing in the Funding section. The updated Funding section is given below and the changes have been highlighted in bold typeface.

Funding

The project was funded by the Swedish Research Council (VR, grant 2012–5167) and the Swedish Research Council for Environment, Agricultural Sciences, and Spatial Planning (FORMAS, grant 2013–627, grant 2018–01423, and grant 2018–0622). **Open access funding provided by Chalmers University of Technology.**

The original article [1] has been corrected.
