# Supplementary material for: Hill-based dissimilarity indices and null models for analysis of microbial community assembly
Source: Microbiome. 2020 Sep 11;8:132. doi: 10.1186/s40168-020-00909-7 (PMC7488682; doi:10.1186/s40168-020-00909-7)
Supplement: Supplementary file 2 — Additional file 1. Dissimilarity indices and null model [file 40168_2020_909_MOESM1_ESM.pdf]

ADDITIONAL FILE 1 – DISSIMILARITY INDICES AND NULL MODEL

Text S1.1: The Jaccard and Bray-Curtis dissimilarity indices

The Jaccard index, originally proposed as an incidence-based similarity measure [1], was calculated as a dissimilarity index using Eq. S1.1.

$$Jaccard = 1 - \frac{Number\ of\ species\ in\ common\ in\ two\ samples}{Total\ number\ of\ species\ in\ two\ samples} \qquad \text{(Eq. S1.1)}$$

The Bray-Curtis index, originally proposed as a similarity measure [2], was calculated as a dissimilarity index using to Eq. S1.2

$$BrayCurtis = 1 - \sum_{i=1}^{S} \min\left(p_{i,1}, p_{i,2}\right) \qquad \text{(Eq. S1.2)}$$

S is the total number of species in the two samples, $p_{i,1}$ and $p_{i,2}$ are the relative abundances of the ith species in samples 1 and 2, respectively.

Text S1.2: Hill-based dissimilarity indices

Eq. S1.3a-b show how Hill diversity numbers (effective numbers) are calculated.

$${}^{q}D = \left(\sum_{i=1}^{S} p_i^q\right)^{1/(1-q)} \qquad \text{(Eq. S1.3a, if q≠1)}$$

$${}^{1}D = exp\left(-\sum_{i=1}^{S}\left(p_i \cdot ln(p_i)\right)\right) \qquad \text{(Eq. S1.3b, if q=1)}$$

D is the Hill diversity number, q is the diversity order, S is the total number of species, and p_i is the relative abundance of the ith species in the sample.

Eq. S1.4a-b show how the Hill numbers for a pair of samples are partitioned into alpha (α), beta (β), and gamma (γ) components.

$${}^{q}D_{\beta} = \frac{{}^{q}D_{\gamma}}{{}^{q}D_{\alpha}} = \frac{\left(\sum_{i=1}^{S}\left(0.5 \cdot p_{i,1} + 0.5 \cdot p_{i,2}\right)^q\right)^{1/(1-q)}}{\left(0.5 \cdot \sum_{i=1}^{S} p_{i,1}^q + 0.5 \cdot \sum_{i=1}^{S} p_{i,2}^q\right)^{1/(1-q)}} \qquad \text{(Eq. S1.4a, if q≠1)}$$

$${}^{1}D_{\beta} = \frac{{}^{1}D_{\gamma}}{{}^{1}D_{\alpha}} = \frac{exp\left(-\sum_{i=1}^{S}\left(\left(0.5 \cdot p_{i,1} + 0.5 \cdot p_{i,2}\right) \cdot \ln\left(0.5 \cdot p_{i,1} + 0.5 \cdot p_{i,2}\right)\right)\right)}{exp\left(-\left(0.5 \cdot \sum_{i=1}^{S} p_{i,1} \cdot \ln\left(p_{i,1}\right) + 0.5 \cdot \sum_{i=1}^{S} p_{i,2} \cdot \ln\left(p_{i,2}\right)\right)\right)} \qquad \text{(Eq. S1.4b, if q=1)}$$

${}^{q}D_{\beta}$ is the beta diversity; ${}^{q}D_{\gamma}$ is gamma diversity, i.e. the Hill number for the two pooled samples; ${}^{q}D_{\alpha}$ is a mean of the alpha diversity in the two samples (see explanation below); and $p_{i,1}$ and $p_{i,2}$ refer to the relative abundances of the ith species in sample 1 and 2, respectively.

Jost [3] showed how diversity could be decomposed into α, β, and γ components for multiple samples. In Eq. S1.4a-b, we only show the equations for a pair of samples; however, the equations are derived from equations 9, 13, and 14 in reference [3]. The α component, which is a mean α-diversity of the two samples, is well described by Chao and Chiu [4] (see equation 5b in reference [4]). They call it "mean effective numbers of species-by-community combinations … per community" [4]. An example of how the calculation is done is shown in **Table S1.1**.

Table S1.1. Hypothetical relative abundance table to show how the γ-diversity and mean α-diversity for two samples are calculated.

Species	**Sample 1**	**Sample 2**	Pooled community
#1	**0.6**	**0**	0.6/2=0.3
#2	**0.4**	**0.7**	(0.4+0.7)/2=0.55
#3	**0**	**0.3**	0.3/2=0.15

The γ-diversity is simply the diversity of the pooled community (Eq. S1.5).

$${}^{q}D_{\gamma} = \left(0.3^q + 0.55^q + 0.15^q\right)^{1/(1-q)} \qquad \text{(Eq. S1.5).}$$

The "mean effective numbers of species-by-community combinations … per community" [4] is given by Eq. S1.6.

$$^{q}D_{\alpha} = \left(\frac{1}{2}\right)\cdot\left(\left(\frac{0.6}{2}\right)^{q} + \left(\frac{0.4}{2}\right)^{q} + \left(\frac{0.7}{2}\right)^{q} + \left(\frac{0.3}{2}\right)^{q}\right)^{1/(1-q)} \quad \text{(Eq. S1.6)}$$

The "species-by-community combination" here refers to **Table S1.1** containing 3 species and 2 samples. The relative abundance for each cell is calculated by dividing the value in the cell by the sum of all values (2 in this case). Then the effective number (diversity) for this species-by-community combination is calculated. The mean effective number is then obtained by dividing this number by 2, since we have two samples in the table. Eq. S1.6 is equivalent to the mean α-diversity in the denominator of Eq. S1.4a. This is shown in Eq. S1.7a-c below. In Eq. S1.7a, $(1/2)^{q}$ is factored out from each term. In Eq. S1.7b, the (1/2) being outside the parenthesis is moved into the parenthesis. In Eq. S1.7c, the equation is simplified and shown to be equivalent to the denominator of Eq. S1.4a.

$$^{q}D_{\alpha} = \left(\frac{1}{2}\right)\cdot\left(\left(\frac{1}{2}\right)^{q}(0.6^{q} + 0.4^{q} + 0.7^{q} + 0.3^{q})\right)^{1/(1-q)} \quad \text{(Eq. S1.7a)}$$

$$^{q}D_{\alpha} = \left(\left(\frac{1}{2}\right)^{1-q}\left(\frac{1}{2}\right)^{q}(0.6^{q} + 0.4^{q} + 0.7^{q} + 0.3^{q})\right)^{1/(1-q)} \quad \text{(Eq. S1.7b)}$$

$$^{q}D_{\alpha} = \left(\left(\frac{1}{2}\right)\cdot(0.6^{q} + 0.4^{q} + 0.7^{q} + 0.3^{q})\right)^{1/(1-q)} \quad \text{(Eq. S1.7c)}$$

Beta diversity ($^{q}D_{\beta}$) can be converted into a dissimilarity index constrained between 0 and 1. Jost [5] showed how β-diversity could be converted to a local overlap measure (dissimilarity subtracted from one). Local means that it takes the perspective of one sample. If two samples each have three species, and only one species is detected in both samples (**Table S1.2**), the local overlap is 1/3 and the corresponding dissimilarity index is 1-1/3=2/3. Written as a dissimilarity index and calculated for any diversity order q, this index is obtained by Eq. S1.8a-b. This set of indices are also referred to as Sørensen-type indices since at diversity order 0, it is identical to the Sørensen dissimilarity index [4, 5].

Beta diversity ($^{q}D_{\beta}$) can also be converted to a regional type of overlap measures. Let us again consider the case with two samples where each has three species and only one species is detected in both (**Table S1.2**). In total, there are five different species in the two samples, and one is found in both samples. Here, the regional overlap is 1/5 and the corresponding dissimilarity is 1-1/5=4/5. Eq. S1.9a-b calculate the regional dissimilarity indices for all diversity orders. This set of indices are also referred to as Jaccard-type indices since at diversity order 0, it is identical to the Jaccard dissimilarity index [4].

Table S1.2. Hypothetical count table to show the difference between local and regional perspective for Hill-based dissimilarity indices (see also Appendix S1 in Chao and Chiu [4]).

Species	Sample 1	Sample 2
#1	1	0
#2	1	0
#3	1	1
#4	0	1
#5	0	1

The local dissimilarity value represents the effective average proportion of OTUs/ASVs in one sample not shared with the other sample. Regional means that the dissimilarity value represents the effective proportion of all OTUs/ASVs that are not present in both samples. In the main paper, we use the local dissimilarity indices, which we call ^{q}d.

$$^{q}d = \frac{({}^{q}D_{\beta})^{(1-q)} - 1}{2^{(1-q)} - 1} \quad \text{(Eq. S1.8a, if q≠1)}$$

$$^{1}d = \frac{ln({}^{q}D_{\beta})}{ln(2)} \quad \text{(Eq. S1.8b, if q=1)}$$

$$^{q}d_{regional} = \frac{1-\left({}^{1}/_{{}^{q}D_{\beta}}\right)^{(1-q)}}{1-\left({}^{1}/_{2}\right)^{(1-q)}} \quad \text{(Eq. S1.9a, if q≠1)}$$

$$^{1}d_{regional} = \frac{ln({}^{q}D_{\beta})}{ln(2)} \quad \text{(Eq. S1.9b, if q=1)}$$

It should be noted that diversity and dissimilarity indices based on Hill numbers also can take phylogenetic- or functional distances between individual species into account [6] using either a phylogenetic tree [7] or a distance matrix as input [8].

Text S1.3: Null model.
With most dissimilarity indices, it is difficult to distinguish between dissimilarity caused by compositional turnover and dissimilarity caused by differences in alpha diversity between two samples [9]. Null models provide a solution to this problem. By random assembly of two samples with pre-defined numbers of species from a regional species pool, a null expectation for the dissimilarity value between the two samples can be generated. If the random assembly process is repeated many times, a distribution for the null expectation is obtained. In this study, we build on the null model by Raup and Crick [10], who used incidence-based data tables. Expressed as a dissimilarity index, their method can be described using Eq. S1.10.

$$RC = \frac{N_{SSexp>SSobs} + 0.5 \cdot N_{SSexp=SSob}}{N_{TOT}} \quad \text{(Eq. S1.10)}$$

RC is the Raup-Crick dissimilarity index, $N_{SSexp>SSobs}$ is the number of randomizations in which the shared species between randomly assembled samples are greater than between observed samples, $N_{SSexp=SSobs}$ is the number of randomizations in which the shared species are equal to the observed samples, and N_{TOT} is the total number of randomizations.

Chase et al. [9] modified the index by normalizing it to a value between -1 and 1 and Stegen et al. [11] extended it to Bray-Curtis dissimilarities. Here, we extend it even further, to all possible Hill-based dissimilarity indices (Eq. S1.11).

$$^{q}RC = \frac{N_{[{}^{q}d_{exp} < {}^{q}d_{obs}]} + 0.5 \cdot N_{[{}^{q}d_{exp} = {}^{q}d_{obs}]}}{N_{TOT}} \quad \text{(Eq. S1.11)}$$

^{q}RC is the Raup-Crick index for any Hill-based dissimilarity of order q, $N_{[qdexp<qdobs]}$ is the number of randomizations in which the dissimilarity between the randomly assembled samples is less than between the observed samples, $N_{[qdexp=qdobs]}$ is the number of randomizations in which the dissimilarities are equal.

With Eq. S1.11, the index is constrained between 0 and 1, where 0 means a lower observed dissimilarity than the null expectation and 1 means a higher observed dissimilarity. Using a significance level (α) of 0.05 [e.g. as in 11], a ^{q}RC value > 0.975 would indicate that the microbial community composition of two samples are statistically more dissimilar to each other than expected by chance and a ^{q}RC < 0.025 would indicate the two samples are more similar to each other than expected by chance.

Text S1.4: Bray-Curtis can be equivalent to ^{1}d under special conditions.
When the following conditions hold for two samples, the ^{1}d dissimilarity is equivalent to the Bray-Curtis dissimilarity.

1. The two samples have the exact same relative abundance distribution.

2. An OTU/ASV detected in both samples has the exact same relative abundance in both samples.

Table S1.3 shows an example when these conditions hold true. Both sample A and B have two OTUs/ASVs with relative abundance of 0.30, two with 0.15, and two with 0.05, which means they have the same relative abundance distribution and condition 1 holds true. We can also see that OTUs/ASVs that are present in both samples (#1, #3, and #4) have the exact same relative abundance in both samples, which means condition 2 holds true.

Table S1.3. Relative abundance table that exemplifies a case when ^{1}d is equal to the Bray-Curtis index.

OTU/ASV	Sample A	Samples B
#1	0.30	0.30
#2	0.30	0
#3	0.15	0.15
#4	0.15	0.15
#5	0.05	0
#6	0.05	0
#7	0	0.30
#8	0	0.05
#9	0	0.05

Under these conditions we can divide the OTUs/ASVs into two sets: those that are shared between the two samples (S_{shared}) and those that are not shared (S_{not}). This equation for the Bray-Curtis dissimilarity can be written as:

$$BrayCurtis = 1 - \sum_{i=1}^{S_{shared}} p_{i,A} \quad \text{(Eq. S1.12a)}$$

$p_{i,A}$ is the relative abundance of OTU/ASV *i* in sample A that is shared with sample B. We could just as well use the OTUs/ASVs in sample B here, since the relative abundance of the shared OTUs/ASVs are the same in both samples.
Eq. S1.12a is equal to:

$$BrayCurtis = \sum_{j=1}^{S_{not}} p_{j,A} \quad \text{(Eq. S1.12b)}$$

$P_{j,A}$ is the relative abundance of OTU/ASV *j* in sample A that is not shared with sample B.
Eq. S1.12a and S1.12b are equivalent since:

$$\sum_{j=1}^{S_{not}} p_{j,A} + \sum_{i=1}^{S_{share}} p_{i,A} = 1 \quad \text{(Eq. S1.12c)}$$

Now, let us have a look at the ^{1}d dissimilarity index. From equations S1.4b and S1.5b, we deduce:

$$^1d = \frac{ln(^1D_\gamma) - ln(^1D_\alpha)}{\ln(2)} \quad \text{(Eq. S1.13a)}$$

$$ln(^1D_\gamma) = -\sum_{i=1}^{S_{shared}} \left(p_{i,A} \cdot \ln(p_{i,A})\right) - 2 \cdot \sum_{j=1}^{S_{not}} \left(\left(\frac{p_{j,A}}{2}\right) \cdot \ln\left(\frac{p_{j,A}}{2}\right)\right) \quad \text{(Eq. S1.13b)}$$

$$ln(^1D_\alpha) = -\sum_{i=1}^{S_{shared}} p_{i,A} \cdot \ln(p_{i,A}) - \sum_{j=1}^{S_{not}} \left(p_{j,A} \cdot \ln(p_{j,A})\right) \quad \text{(Eq. S1.13c)}$$

$$ln(^1D_\gamma) - ln(^1D_\alpha) = \sum_{j=1}^{S_{not}} \left(p_{j,A} \cdot \ln(2)\right) \quad \text{(Eq. S1.13d)}$$

$$^1d = \frac{ln(^1D_\gamma) - ln(^1D_\alpha)}{ln(2)} = \sum_{j=1}^{S_{not}} \left(p_{j,A}\right) \quad \text{(Eq. S1.13e)}$$

Thus, Eq. S1.12b and S1.13e show that ^{1}d is equivalent to the Bray-Curtis dissimilarity index under the special conditions described above.

References

1. Jaccard P: **The distribution of the flora in alpine zone**. *The New Phytologist* 1912, **11**(2):37-50.
2. Bray JR, Curtis JT: **An ordination of the upland forest communities of Southern Wisconsin**. *Ecological Monographs* 1957, **27**(4):325-349.
3. Jost L: **Partitioning diversity into independent alpha and beta components**. *Ecology* 2007, **88**(10):2427-2439.
4. Chao A, Chiu C-H: **Bridging the variance and diversity decomposition approaches to beta diversity via similarity and differentiation measures**. *Methods in ecology and evolution* 2016, **7**(8):919-928.
5. Jost L: **Entropy and diversity**. *OIKOS* 2006, **113**(2):363-375.
6. Chao A, Chiu C-H, Jost L: **Unifying species diversity, phylogenetic diversity, functional diversity, and related similarity and differentiation measures through Hill numbers**. *Annu Rev Ecol Evol Syst* 2014, **45**:297-324.
7. Chiu CH, Jost L, Chao A: **Phylogenetic beta diversity, similarity, and differentiation measures based on Hill numbers**. *Ecological Monographs* 2014, **84**(1):21-44.
8. Chiu CH, Chao A: **Distance-based functional diversity measures and their decomposition: a framework based on Hill numbers**. *PLoS One* 2014, **9**(7):e100014.
9. Chase JM, Kraft NJB, Smith KG, Vellend M, Inouye BD: **Using null models to disentangle variation in community dissimilarity from variation in α-diversity**. *Ecosphere* 2011, **2**(2):24.
10. Raup DM, Crick RE: **Measurement of faunal similarity in paleontology**. *Journal of Paleontology* 1979, **53**(5):1213-1227.
11. Stegen JC, Lin X, Fredrickson JK, Chen X, Kennedy DW, Murray CJ, Rockhold ML, Konopka A: **Quantifying community assembly processes and identifying features that impose them**. *ISME Journal* 2013, **7**(11):2069-2079.
