# Supplementary material for: Hill-based dissimilarity indices and null models for analysis of microbial community assembly
Source: Microbiome. 2020 Sep 11;8:132. doi: 10.1186/s40168-020-00909-7 (PMC7488682; doi:10.1186/s40168-020-00909-7)
Supplement: Supplementary file 3 — Additional file 2. Bioinformatics pipelines and consensus algorithm [file 40168_2020_909_MOESM2_ESM.pdf]

ADDITIONAL FILE 2 – BIOINFORMATICS PIPELINES AND CONSENSUS ALGORITHM

Text S2.1: Description of bioinformatics pipeline settings.

Scripts used to run the bioinformatics pipelines can be found at https://github.com/omvatten/amplicon_sequencing_pipelines. Bash scripts (ending with .sh) were executed from the terminal window of a Linux computer by typing:
bash "script_name.sh"
R scripts (ending with .R) were executed in RStudio [1, 2]. Python scripts (ending with .py) were executed in Spyder (https://ww w.spyder-ide.org/) unless they were called within Bash scripts.

DADA2
The R script DADA2_pipeline.R was run in RStudio. The script was run four times with different settings to generate four count tables for each data set (**Table S2.1**).

The filterAndTrim command was run with truncation lengths (truncLen) of 220 (forward reads) and 150 (reverse reads) for the AGS samples, and 220 and 100 for the MFC samples. The maximum allowed errors (maxEE) in a read was 2 and 5 for the AGS samples and 2 and 2 for the MFC samples. The maximum number of Ns (maxN) was set to 0 for both data sets.

The dada function was run with the OMEGA_A parameter set to either 1e-40 to generate fewer ASVs or 1e-10 to generate more ASVs. The samples were either processed one-by-one or in pooled mode by setting the pool parameter to either FALSE or TRUE). Chimeras were removed using the removeBimeraDenovo command with method="consensus".

To reformat the count table generated with DADA2 to fit the input format of qdiv, a Python script was used (DADA2_convert_to_qDiv_input.py). The script was run in Spyder.

Table S2.1. Summary of settings used in the DADA2 pipelines. The AGS and MFC pipeline numbers corresponds to the numbers for the count tables in Fig. S2.1.

	AGS pipelines				MFC pipelines			
**Command**	**1**	**2**	**3**	**4**	**1**	**2**	**3**	**4**
filterAndTrim(truncLen=)	(220,150)				(220,100)			
filterAndTrim(maxEE=)	(2,5)				(2,2)			
dada(OMEGA_A=)	1e-10	1e-40	1e-10	1e-40	1e-10	1e-40	1e-10	1e-40
dada(pool=)	TRUE	TRUE	FALSE	FALSE	TRUE	TRUE	FALSE	FALSE

Deblur
The Deblur pipeline was run in Qiime2 [3]. The commands executed in a terminal window on a Linux computer are listed in the file Deblur_via_QIIME2.pdf.

In the Deblur pipeline, the sequences were first truncated to 250 bp and merged using vsearch join-pairs in Qiime2. The AGS samples were merged with default settings. For the MFC samples, the maximum number of differences in the sequence alignment (maxdiffs) parameter was changed from 10 (default value) to 50. The sequences from the MFC run had lower quality and changing the maxdiffs parameter allowed more reads to be merged. The deblur algorithm was run with trim-length set to 250 bp.

USEARCH
The USEARCH pipelines were run as bash scripts on a Linux computer. Two scripts were developed, one for pooled samples and one for samples processed separately. The scripts were implemented in a terminal window as:
bash USEARCH_pipeline_pooled_samples.sh

bash USEARCH_pipeline_nonpooled_samples.sh

The scripts were run five times for each data set to generate five count tables (**Table S2.2**). For three count tables, the unoise3 command was used to generate ASVs and for two count tables, the cluster_otus command was used to cluster OTUs. The fastq_mergepairs command was run with 200 and 270 as the minimum and maximum merge lengths, respectively. The fastq_maxdiffs and fastq_pctid parameters were set to 10 and 90 for the AGS samples and 50 and 80 for the MFC samples. Quality filtering was done using a maxee threshold of 0.2 under stringent settings and 1 under more relaxed settings. ASVs were inferred with the unoise3 command using a minsize parameter of 8 (stringent settings) and 4 (relaxed settings). Using UPARSE and the cluster_otus command, a minsize of 2 was used. Merged reads were mapped to the OTUs/ASVs using the otutab command with an id setting of 0.99 for ASVs and 0.97 for OTUs.

In the USEARCH pipeline, we initially experienced an error when calling the fasta file zotus_d_p_e_s.fa, containing the ASV sequences generated with the unoise3 command. The error disappeared when the names of the sequences where changed from ZotuX (where X is a number) to OtuX. The Python script, USEARCH_change_Zotu_to_Otu.py, makes this change and is called inside the bash scripts.

When the USEARCH pipeline was run in nonpooled mode, i.e. each sample was processed separately, a Python script (USEARCH_merge_nonpooled_tables.py) was used to merge the data into one count table. This Python script was executed in Spyder after the bash script had completed.

Table S2.2. Summary of settings used in the USEARCH pipelines. The AGS and MFC pipeline numbers corresponds to the numbers for the count tables in Fig. S2.1.

	**AGS pipelines**					**MFC pipelines**				
**Command**	**6**	**7**	**8**	**9**	**10**	**6**	**7**	**8**	**9**	**10**
Bash script										
USEARCH_pipeline_pooled.sh	X	X		X		X	X		X	
USEARCH_pipeline_nonpooled.sh			X		X			X		X
fastq_mergepairs	-fastq_maxdiffs 10<br>-fastq_pctid 90					-fastq_maxdiffs 50<br>-fastq_pctid 80				
fastq_filter -fastq_maxee	1	0.2	1	1	1	1	0.2	1	1	1
unoise3 -minsize	4	8	4			4	8	4		
cluster_otus -minsize				2	2				2	2
otutab -id	0.99	0.99	0.99	0.97	0.97	0.99	0.99	0.99	0.97	0.97

Mothur
In Mothur, we followed the MiSeq SOP (https://www.mothur.org/wiki/MiSeq_SOP, accessed 2019-04-23).

Text S2.2: Algorithm for inferring consensus table from two or more count tables.
This algorithm works for ASV count tables generated using denoiser pipelines (e.g. DADA2, UNOISE, Deblur). It should not be used for count tables generated using OTU clustering since the representative OTU sequences generated with different pipelines cannot be directly compared.

Step 1 - Identify amplicon sequence variants (ASVs) that are found in all count tables.
The algorithm identifies sequences that are identical in the different count tables. Some pipelines, such as Deblur [4], requires the user to truncate all sequences to a common length. If a truncated sequence is compared to a full-length sequence generated with e.g. DADA2, the two sequences will appear different even though they actually represent the same ASV. To account for this, the algorithm has an option to classify two sequences as identical if one can be found within another. For example,

in the case below Seq 1 can be considered identical to Seq 2 even though Seq 2 is longer. The algorithm keeps the longer sequence (Seq 2) as representative for the ASV in the consensus table.
Seq 1: 'AATTCGACCGGT'
Seq 2: 'AATTCGACCGGTATG'
It should be noted; however, that this can introduce errors in the data. Sequences of different lengths, where one sequence can be found within the other, may represent two different biological entities. Therefore, this option should be used with care.

Step 2 - Decide which count table to use as basis for the consensus table.
In step 1, the ASVs common to all count tables were determined. Here, the fractions of reads associated with these ASVs are calculated in each count table. The count table with the highest fraction belonging to the common ASVs is chosen. It is also possible for the user to specify which count table to keep.

Step 3 – Subset the chosen count table.
In the chosen count table, all ASVs belonging to the common ASVs are retained. The rest of the ASVs are filtered out.

Text S2.3: Comparison of 12 count tables – alpha diversity
Eleven count tables were generated using DADA2, Deblur, USEARCH (UNOISE or UPARSE), and Mothur. One consensus table was also generated based on two count tables from DADA2 and UNOISE. **Fig. S2.1** shows information about the generated count tables, including how the sequences were processed and the numbers of reads and OTUs/ASVs. Sequence reads were processed sample-by-sample (separate) or by pooling the reads in all samples (pooled). Relaxed quality filtering thresholds resulting in a higher number of OTUs/ASVs (high) or stringent settings resulting in fewer OTUs/ASVs (low) were used in some of the pipelines. Deblur resulted in the lowest number of ASVs/OTUs in both the AGS- and MFC experiments and Mothur resulted in the highest. There was a large span in the number of inferred OTUs/ASVs for different pipelines. In the AGS experiment the number ranged from 690 to 4055 and in the MFC experiment the span was 1800 to 6457. During analysis of dissimilarities, all count tables were rarefied (without replacement) to the read count in the sample with the lowest number of reads. This resulted in count tables with 223 692 to 321 060 reads per sample in the AGS data set and 15 825 to 35 680 in the MFC data set **(Fig. S2.2)**. The order of magnitude difference in reads per sample for the two data sets was caused by a higher number of total reads, a lower number of samples, and a more even sequencing depth in the AGS data set.

Previous studies comparing bioinformatics pipelines for high-throughput sequencing of marker-genes have found large differences in alpha diversity estimates [5, 6]. Here, we observed that both the pipeline and the input parameter values chosen by the user affected the number of inferred OTUs/ASVs as well as the number of reads mapped to these. With real samples of unknown composition, it is difficult to choose which pipeline and which settings to use for the analysis. A way to analyze the count tables generated with different pipelines is to use rarefaction curves (**Fig. S2.3-4**) and octave plots (**Fig. S2.5-6**) [7]. Continuously increasing rarefaction curves and inflated numbers of singletons in the octave plots are most likely consequences of incorrect OTUs/ASVs, e.g., caused by sequencing errors remaining in the count tables after bioinformatic processing [8]. This can, for example, be observed in count table #11 (**Fig. S2.3-6**). Several pipelines filter out low abundant OTUs/ASVs because they are likely to be incorrect, and for that reason several count tables lack singletons, e.g. count table #8 (**Fig. S2.5-6**). This also results in rarefaction curves rapidly reaching a horizontal asymptote **(Fig. S2.3-4)**.

Despite large differences in the number of low-abundant OTUs/ASVs in the count tables, abundance-based diversity metrics were similar. Pielou's evenness index, ^{1}D and ^{2}D (Hill diversity of order 1 and

2) were not much affected by pipeline (**Fig. S2.7**). The count tables generated with DADA2 in pooled mode for the MFC data set were exceptions. These pipelines had much fewer reads being mapped to the category of samples called "Anode acetate" (**Fig. S2.8**), which resulted in those samples having higher evenness and diversity than in the other pipelines (**Fig. S2.7**).

Text S2.4: Comparison of 12 count tables – beta diversity

Having seen differences in the number of OTUs/ASVs and read counts in count tables generated with different bioinformatics pipelines, we asked if dissimilarity matrices calculated from the count tables would show the same patterns in the data. Matrices of pairwise dissimilarities between samples are typically used to explore microbial communities. First, we investigated the similarity of dissimilarity matrices generated using different indices and count tables. The matrices were compared using Mantel's permutation test [9] with the Pearson correlation coefficient (r) or the Spearman rank correlation coefficient (ρ) as test statistic. For all pairwise comparisons between dissimilarity matrices, there was a statistically significant correlation (p=0.001, 999 permutations), and the correlation coefficients ranged from 0.71 to 1.00. Although the dissimilarity matrices were highly correlated with each other, there were some differences. To visualize these differences, a principal coordinate analysis (PCoA) of the dissimilarities (measured as 1-r or 1-ρ) between dissimilarity matrices was carried out **(Fig. S2.9-10)**. Each point in **Fig. S2.9** and **S2.10** represents a dissimilarity matrix calculated from a rarefied count table. The matrices tended to separate by index. Incidence-based indices (^{0}d and Jaccard) were more scattered and clearly separated from relative-abundance based indices (^{1}d, ^{2}d, Bray-Curtis).

The ability of the different count tables to distinguish between sample groups in the experimental data was also tested. The AGS data set was more challenging than the MFC data set because most taxa were shared between different samples. Therefore, the AGS data set with the three sample categories, the inoculum, reactor 1 (R1), and reactor 2 (R2), was used in the analysis. Using permanova, statistically significant separation between the sample groups was found with all count tables and dissimilarity indices (p=0.001, 999 permutations). A PCoA for the consensus table and the ^{0}d index is shown in **Fig. S2.11**.

Text S2.5: Dissimilarity between replicates

A consensus table was generated for each data set using count tables #3 and #6 as input. The dissimilarity between replicate samples for the consensus table was compared to the dissimilarity between replicates for the input count tables. The results are shown in **Fig. S2.12.**

Dissimilarity between replicates at low diversity order is typically a consequence of undersampling. The effect of sequencing depth of the coverage of ASVs was tested for the AGS data set. A sequencing depth of almost 4 million reads was needed to cover 99% of the 919 ASVs found in the consensus count table (**Fig. S2.13**).

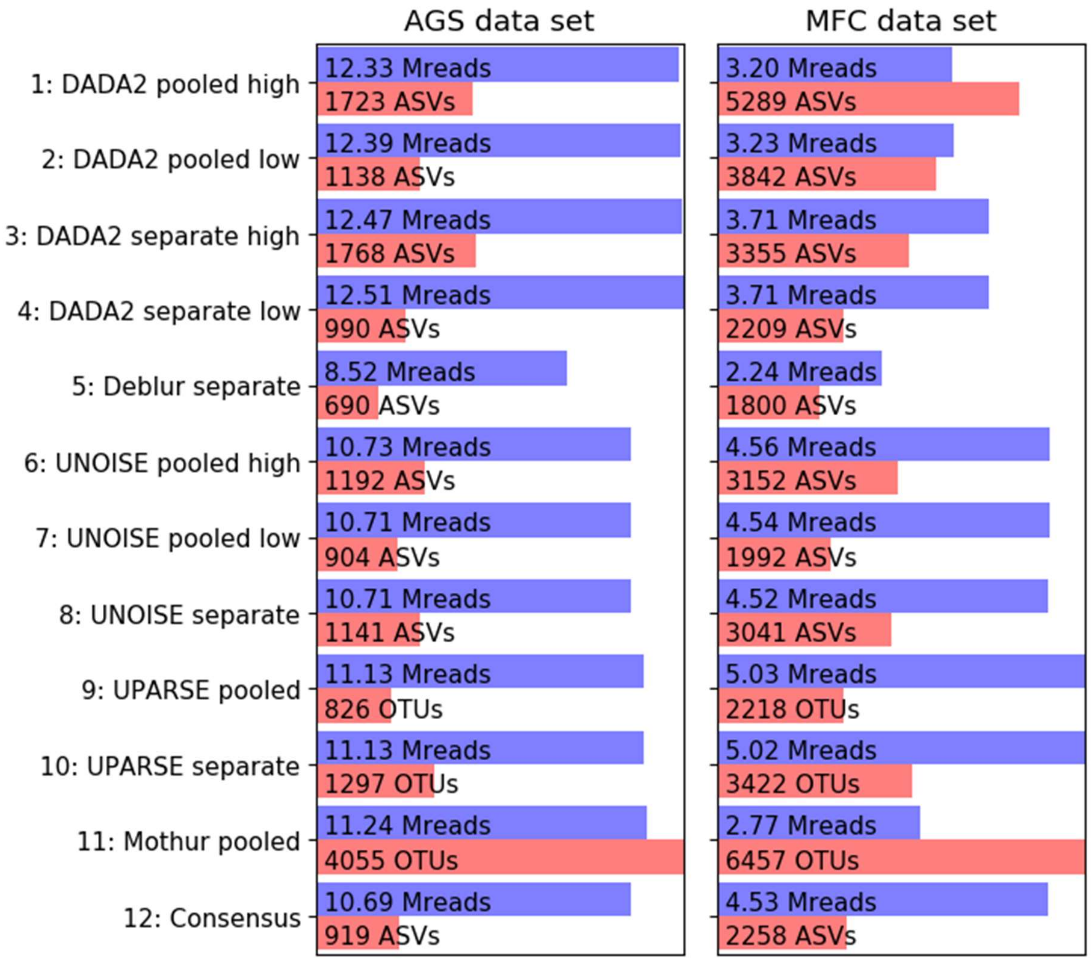

Fig. S2.1. Count tables generated using different bioinformatics pipelines. The total number of reads (in million reads, Mreads) and the total number of detected OTUs/ASVs are shown. The lengths of the bars show the numbers for the count tables in relation to each other within each data set. The consensus count tables are inferred based on count tables#3 and #6 in both data sets.

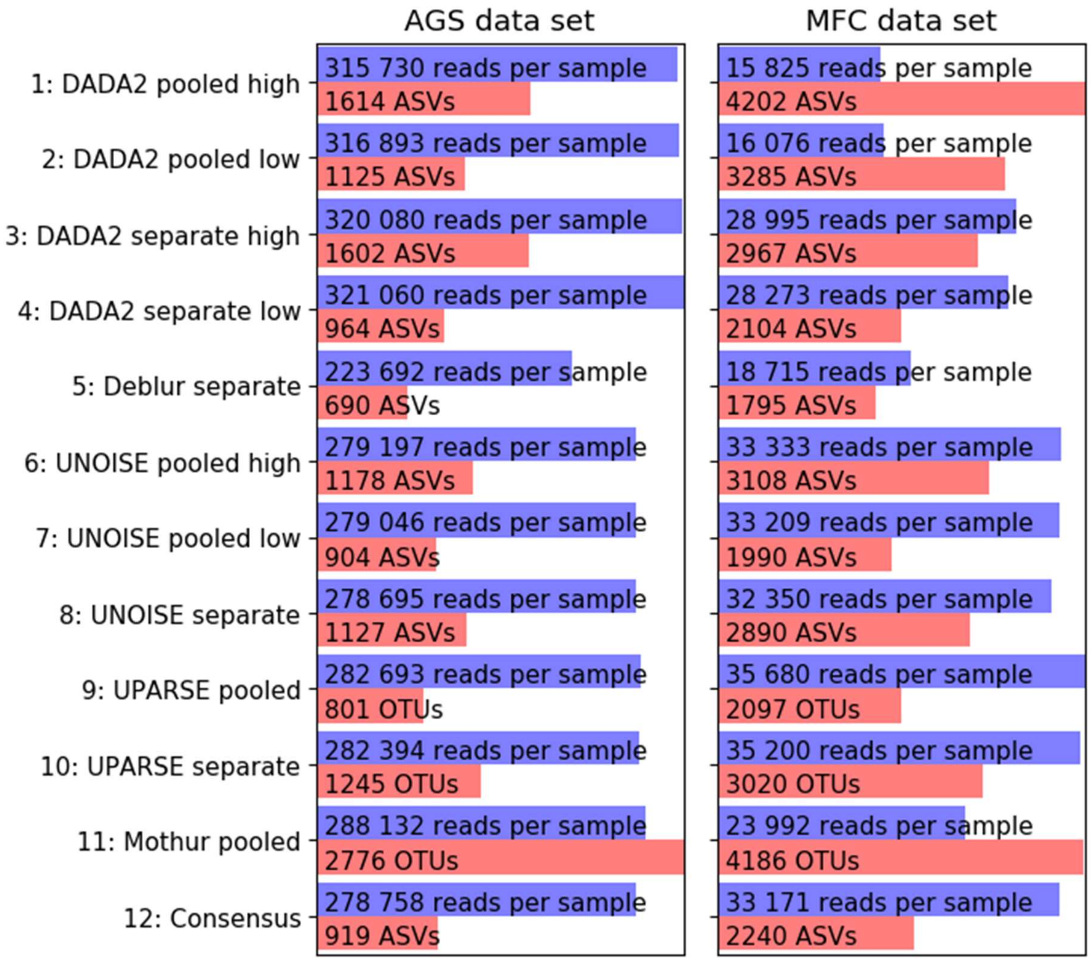

Fig. S2.2. Number of reads per sample and the total number of OTUs/ASVs in the rarefied count tables. The lengths of the bars show the numbers for the count tables in relation to each other within each data set. The consensus count tables are inferred based on count tables#3 and #6 in both data sets.

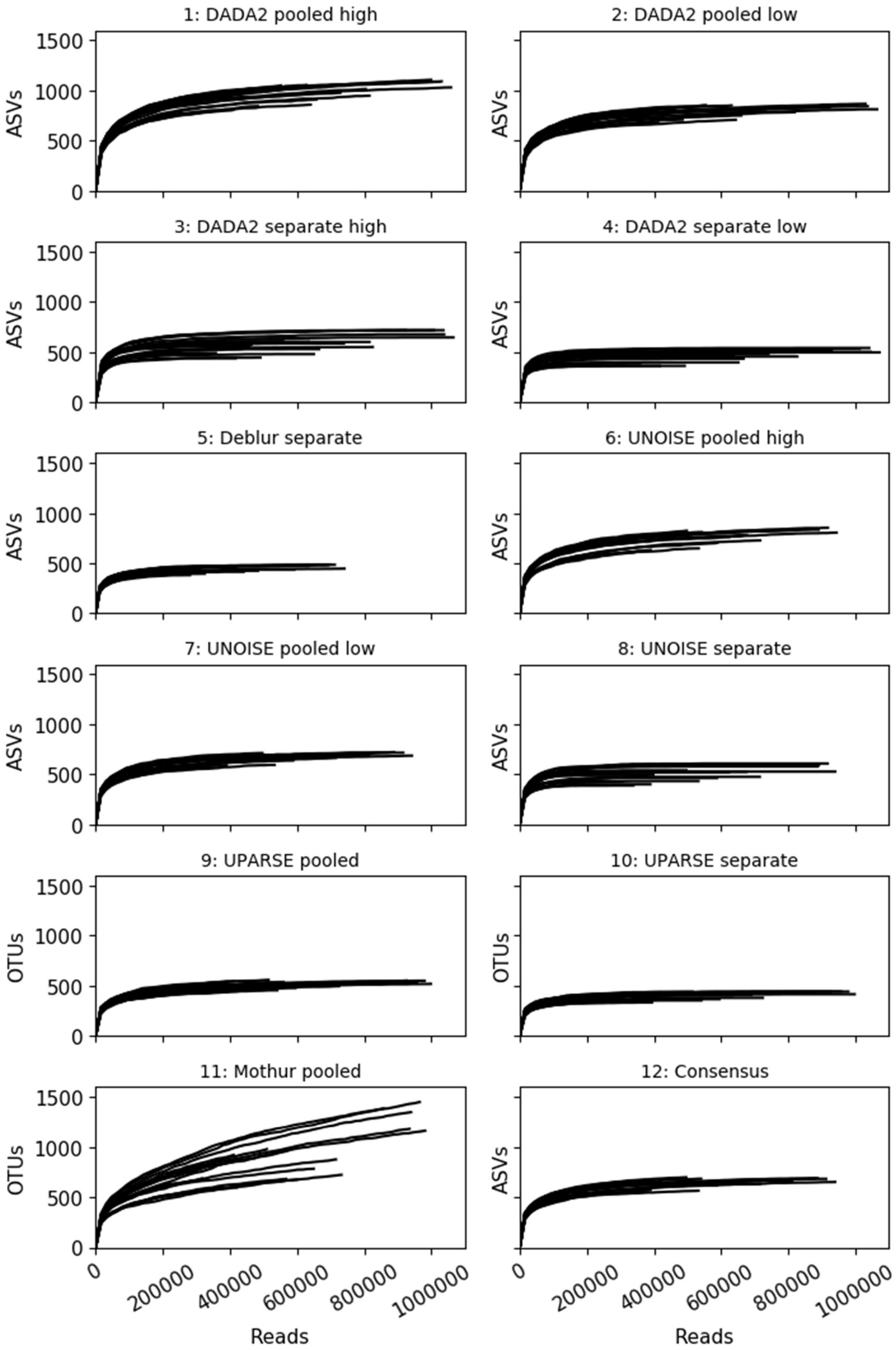

Fig S2.3. Rarefaction curves for the count tables generated with the AGS data set.

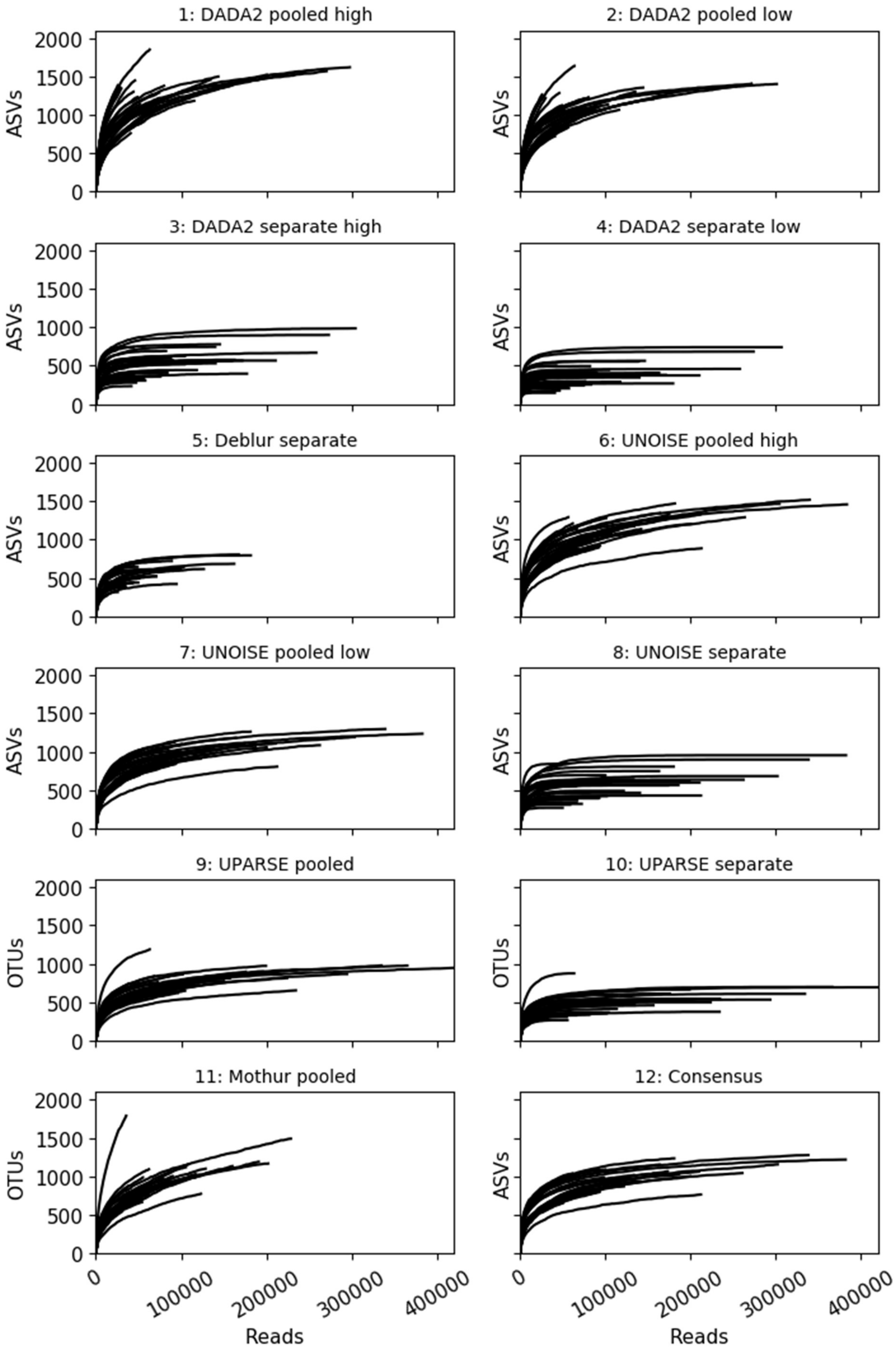

Fig S2.4. Rarefaction curves for the count tables generated with the MFC data set.

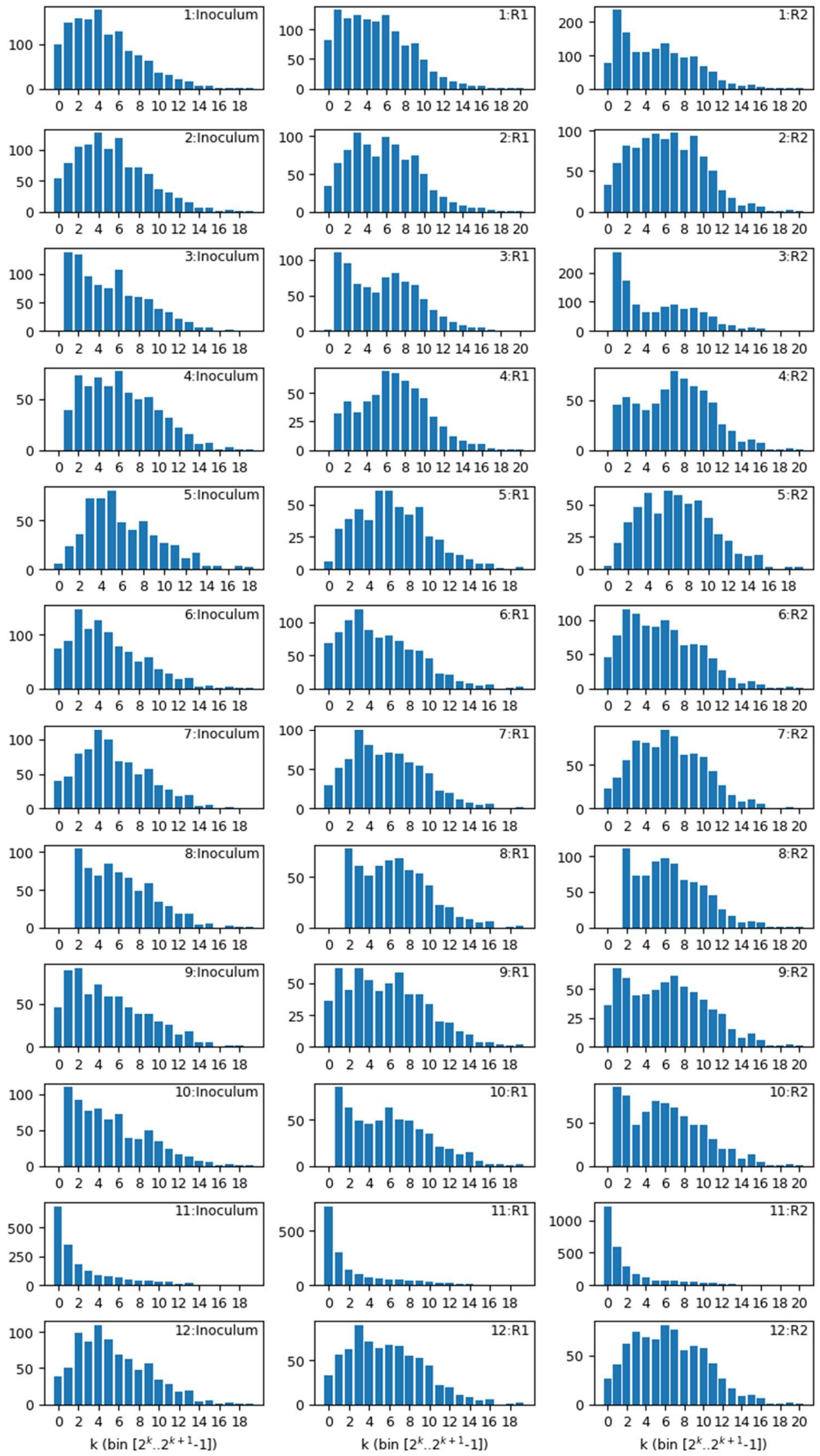

Fig. S2.5. Octave plots for each sample category in the AGS data set. The count table (indicated by number) and sample category used to generate the plot are shown in each panel. The x-axis shows bins with read counts. The y-axis shows the number of OTUs/ASVs having read counts associated with each bin.

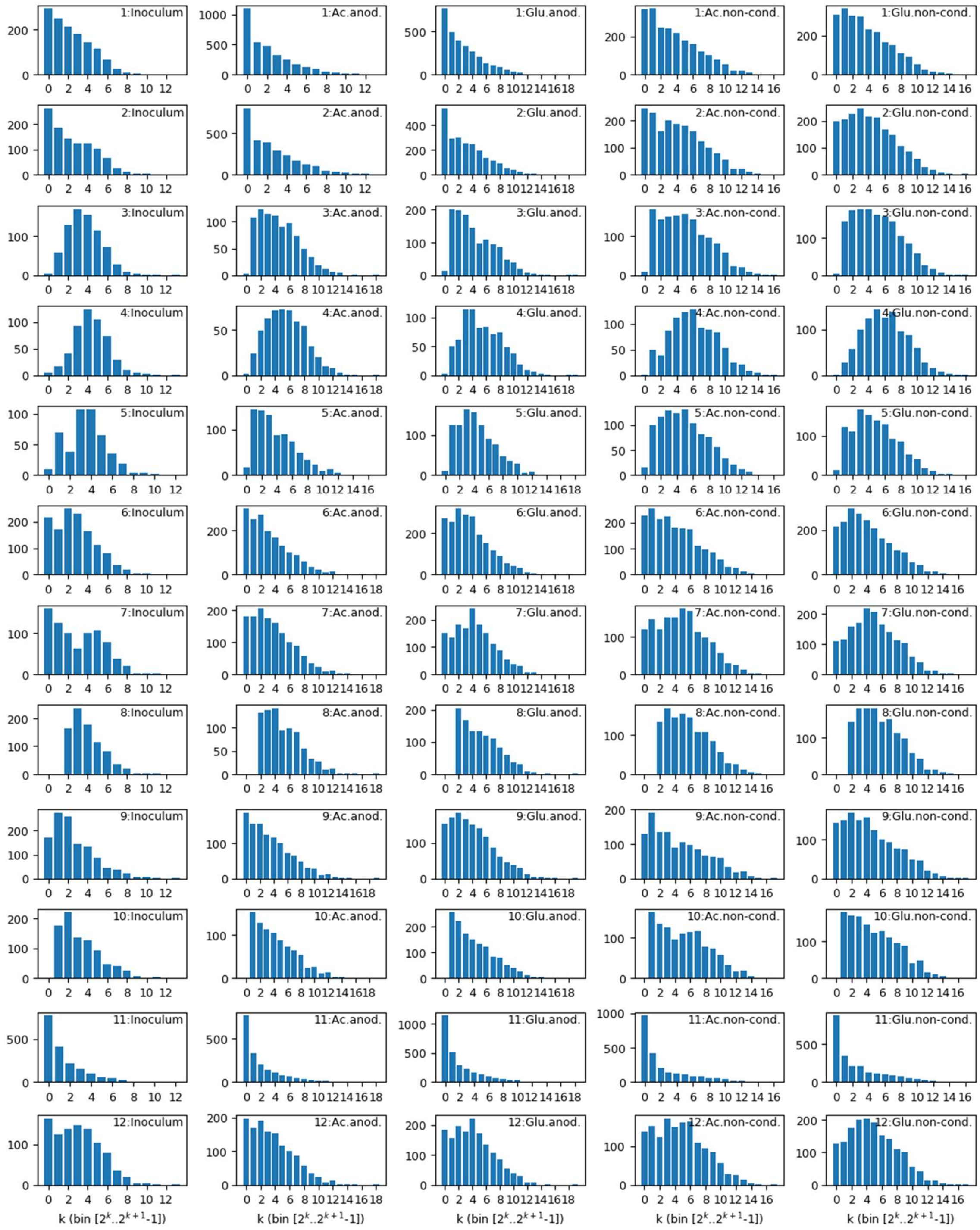

Fig. S2.6. Octave plots for each sample category in the MFC data set. The count table (indicated by number) and sample category used to generate the plot are shown in each panel. The x-axis shows bins with read counts. The y-axis shows the number of OTUs/ASVs having read counts associated with each bin.

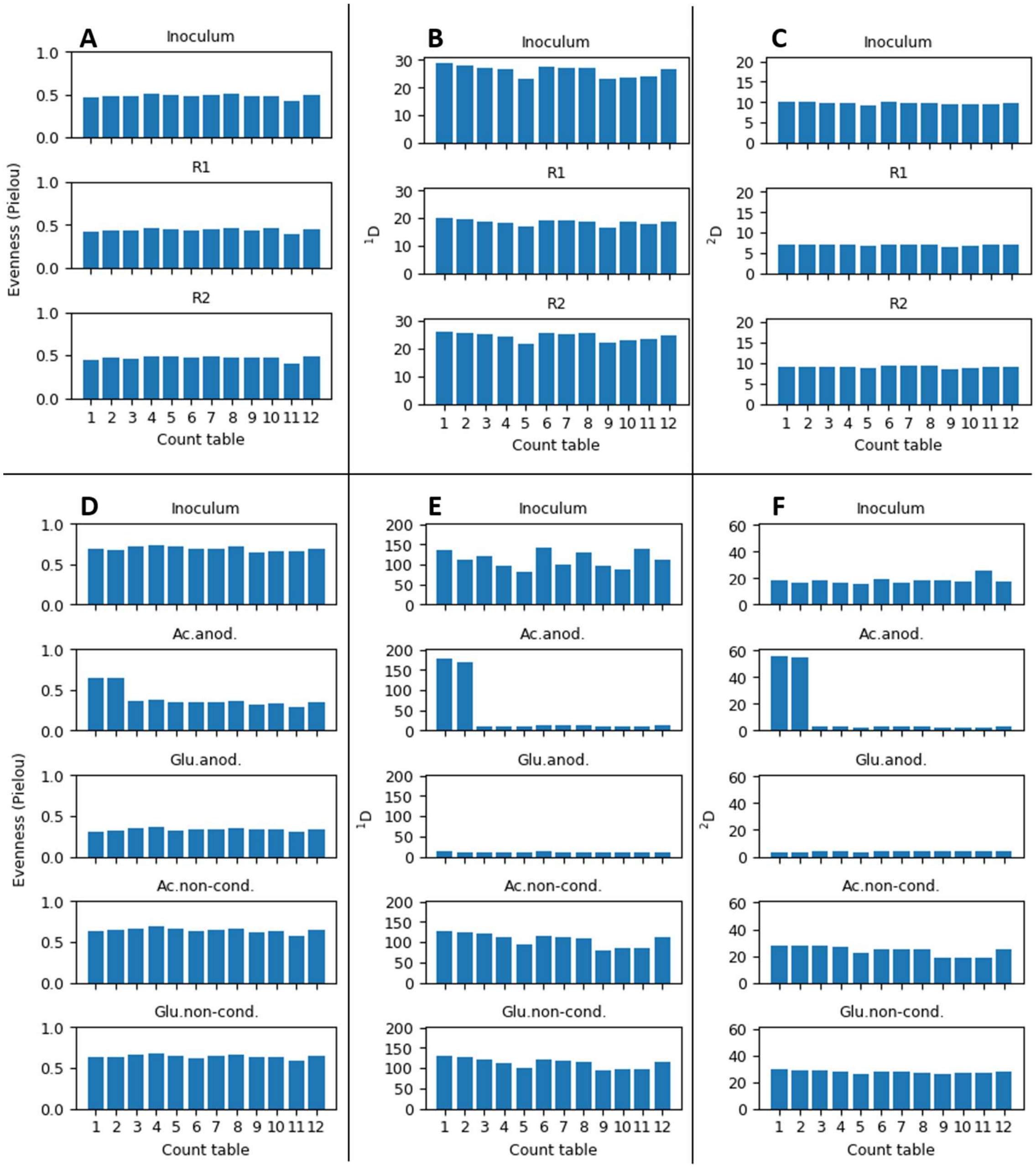

Fig. S2.7. Pielou's evenness (A, D), Hill diversity of order 1 (B, E) and 2 (C, F) for the AGS (A-C) and the MFC (D-F) data sets.

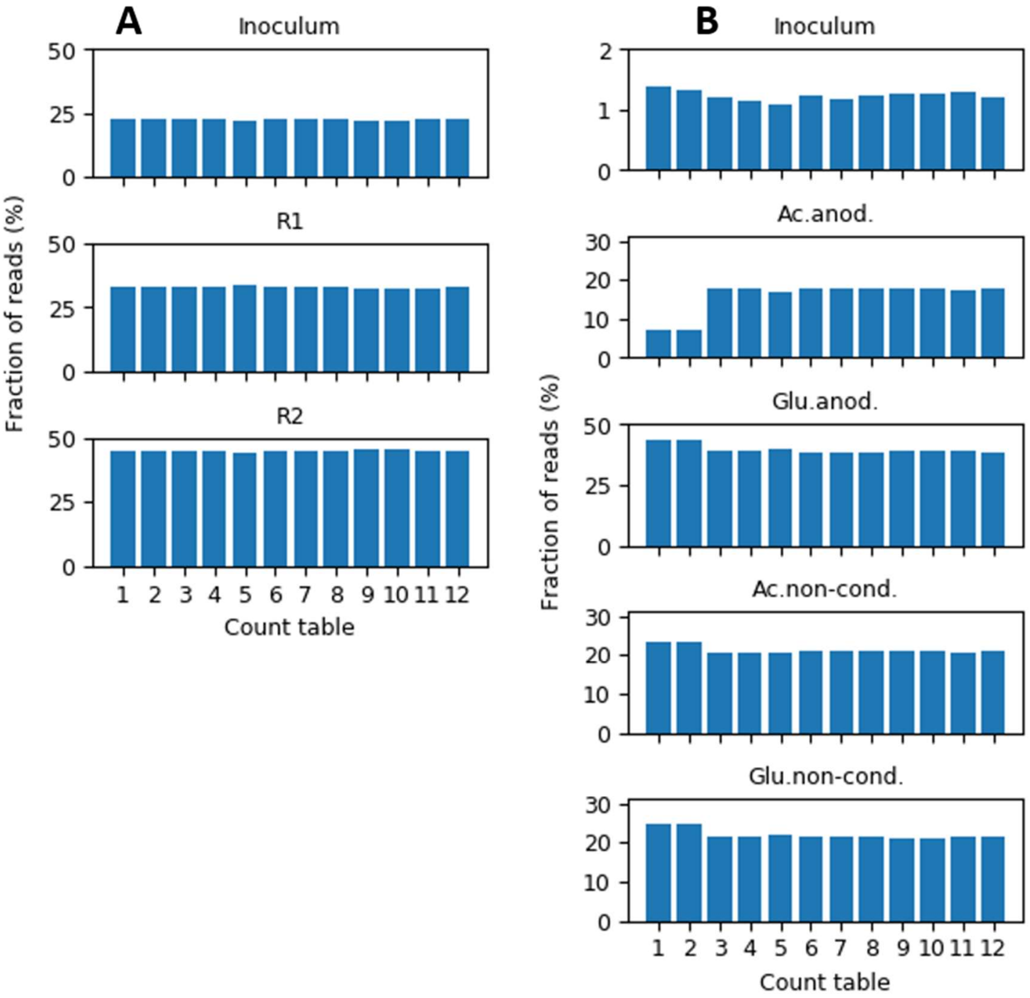

Fig. S2.8. Fraction of reads mapped to the different sample categories in the AGS (A) and MFC (B) data sets.

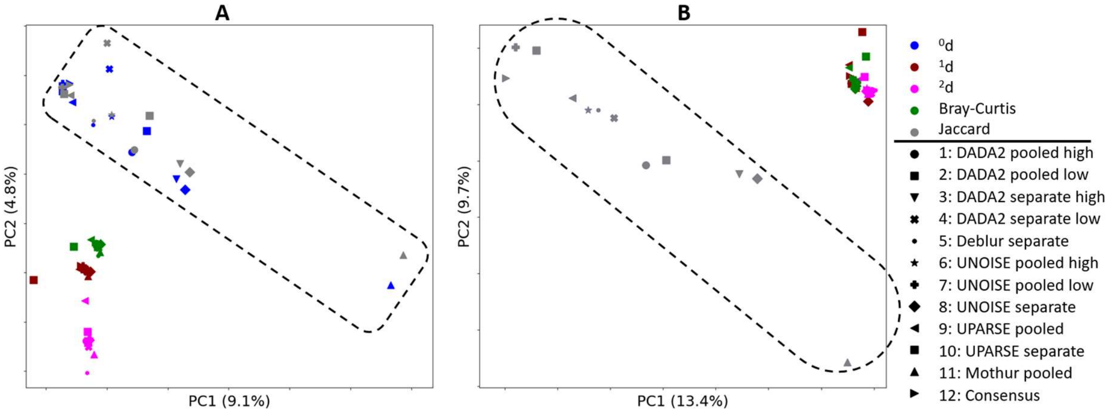

Fig. S2.9. PCoA based on correlations between dissimilarity matrices from the AGS data set. Pearson's r (A) and Spearman's ρ (B) subtracted from one were used a dissimilarity values to generate the dissimilarities between dissimilarity matrices. The points encircled by a dashed line are dissimilarity matrices generated with incidence-based indices (^{0}d and Jaccard).

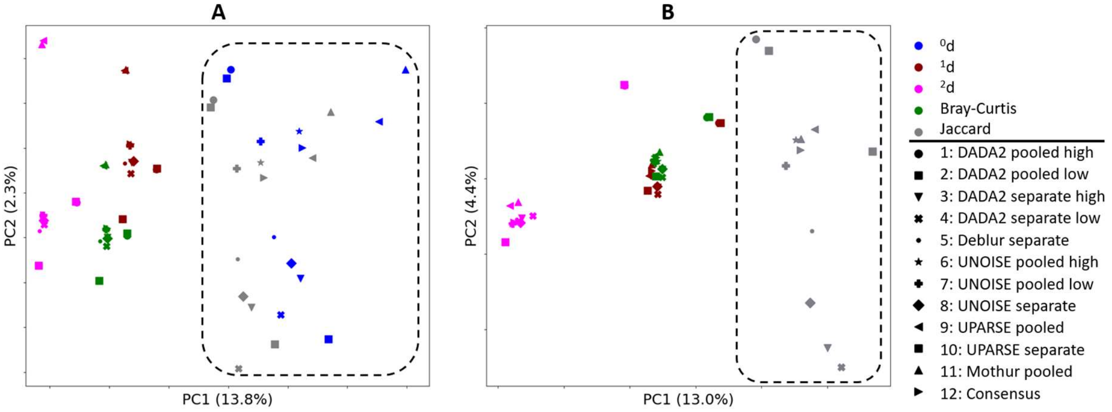

Fig. S2.10. PCoA based on correlations between dissimilarity matrices from the MFC data set. Pearson's r (A) and Spearman's ρ (B) subtracted from one were used a dissimilarity values to generate the dissimilarities between dissimilarity matrices. The points encircled by a dashed line are dissimilarity matrices generated with incidence-based indices (^{0}d and Jaccard).

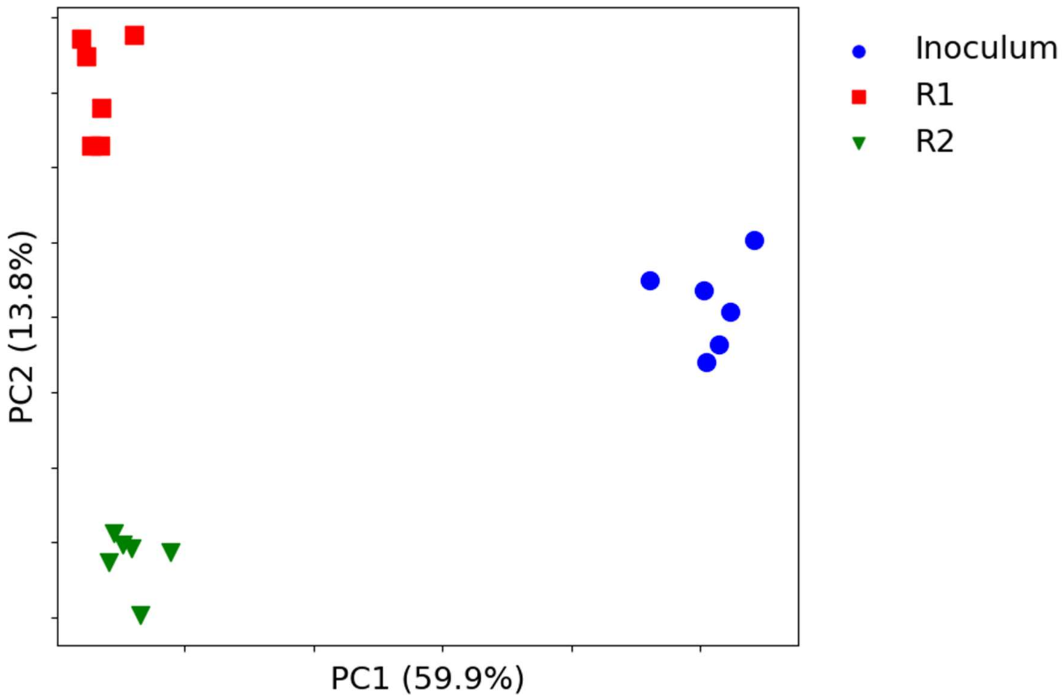

Fig. S2.11. PCoA based on ^0d for the consensus table from the AGS data set. The three sample categories are clearly separated.

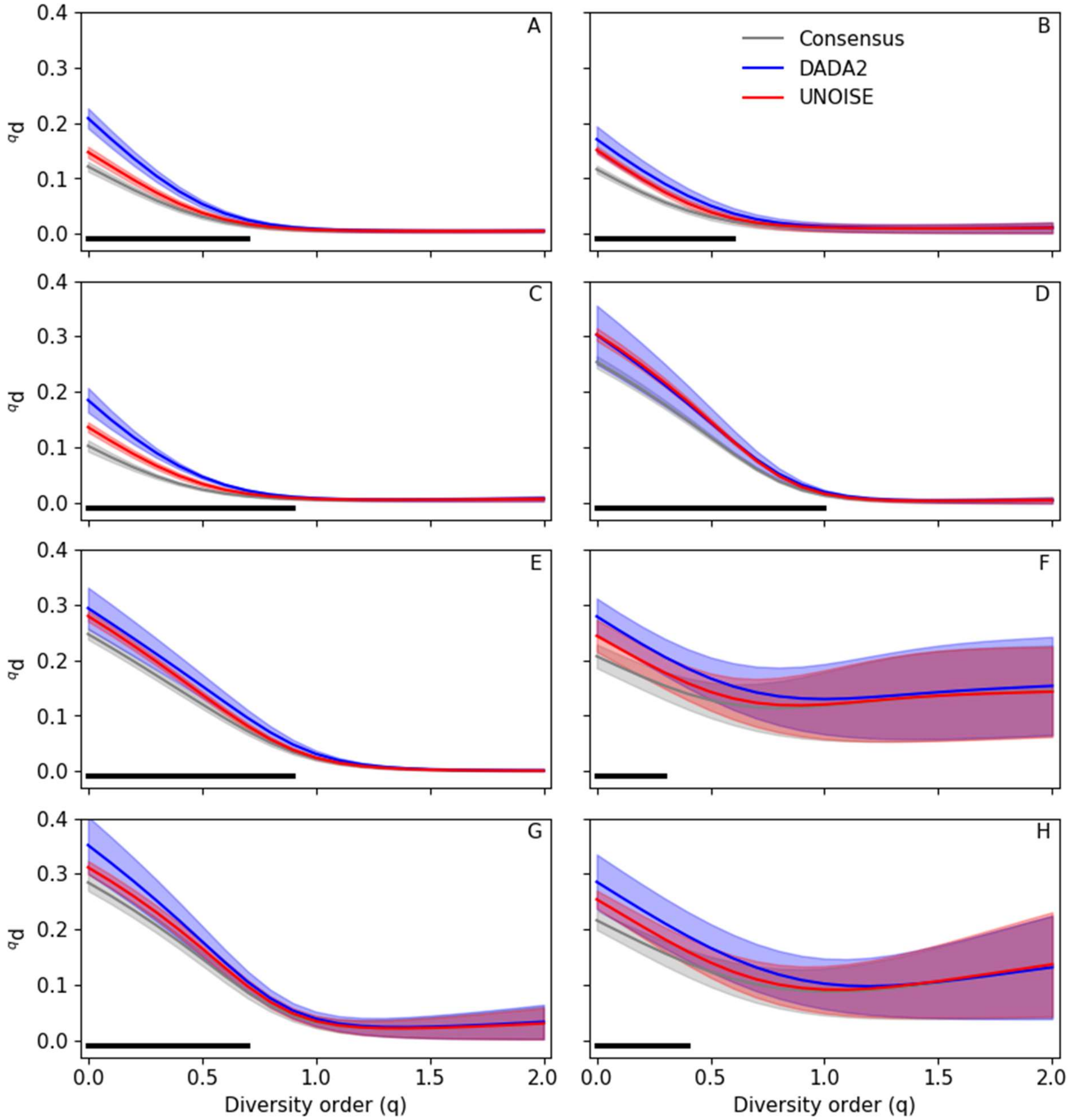

Fig. S2.12. Dissimilarity between replicates. The inoculum (A), R1 (B), and R2 (C) samples from the AGS data set, and the technical replicates (D), the acetate-fed anode biofilm (E), the acetate-fed biofilm on non-conductive surface (F), the glucose-fed anode biofilm (G), and the glucose-fed biofilm on non-conductive surface (H) samples from the MFC data set are shown in the panels. Shaded regions show standard deviations. Consensus is count table #12, DADA2 and UNOISE refer to count tables #3 and #6, respectively (see Fig. S2). The black horizontal line marks diversity orders with significantly lower dissimilarity between replicates for the consensus table in comparison to the other two count tables ($p < 0.05$, n=15, Student's t-test).

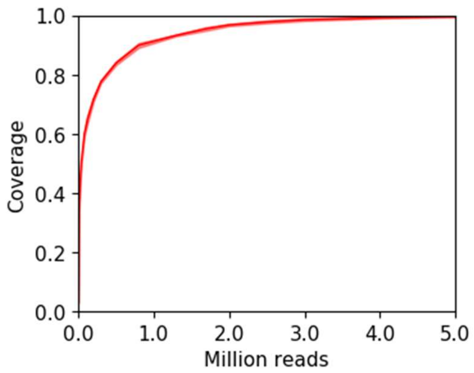

Fig. S2.13. Coverage (i.e. fraction of detected ASVs) as a function of sequencing depth for the simulation using the AGS data set.

References

1. RStudio Team: **RStudio: Integrated Development for R**. PBC, Boston, MA; 2020.
2. R Core Team: **R: A language and environment for statistical computing. R Foundation for Statistical Computing**. Vienna, Austria; 2017.
3. Bolyen E, Rideout JR, Dillon MR, Bokulich NA, Abnet CC, Al-Ghalith GA, Alexander H, Alm EJ, Arumugam M, Asnicar F *et al*: **Reproducible, interactive, scalable and extensible microbiome data science using QIIME 2**. *Nature Biotechnology* 2019, **37**(8):852-857.
4. Amir A, McDonald D, Navas-Molina JA, Kopylova E, Morton JT, Zech Xu Z, Kightley EP, Thompson LR, Hyde ER, Gonzalez A *et al*: **Deblur Rapidly Resolves Single-Nucleotide Community Sequence Patterns**. *mSystems* 2017, **2**(2).
5. Allali I, Arnold JW, Roach J, Cadenas MB, Butz N, Hassan HM, Koci M, Ballou A, Mendoza M, Ali R *et al*: **A comparison of sequencing platforms and bioinformatics pipelines for compositional analysis of the gut microbiome**. *BMC Microbiol* 2017, **17**(1):194.
6. Nearing JT, Douglas GM, Comeau AM, Langille MGI: **Denoising the Denoisers: an independent evaluation of microbiome sequence error-correction approaches**. *PeerJ* 2018, **6**:e5364.
7. Edgar RC, Flyvbjerg H: **Octave plots for visualizing diversity of microbial OTUs**. *bioRxiv* 2018:389833.
8. Edgar RC, Flyvbjerg H: **Alpha diversity metrics for noisy OTUs**. *bioRxiv* 2018:434977.
9. Mantel N: **The Detection of Disease Clustering and a Generalized Regression Approach**. *Cancer Research* 1967, **27**(2 Part 1):209-220.
