# Supplementary material for: Hill-based dissimilarity indices and null models for analysis of microbial community assembly
Source: Microbiome. 2020 Sep 11;8:132. doi: 10.1186/s40168-020-00909-7 (PMC7488682; doi:10.1186/s40168-020-00909-7)
Supplement: Supplementary file 4 — Additional file 3. Experimental details [file 40168_2020_909_MOESM3_ESM.pdf]

Oskar Modin, Raquel Liébana, Soroush Saheb-Alam, Britt-Marie Wilén, Carolina Suarez, Malte Hermansson, Frank Persson

## ADDITIONAL FILE 3 – EXPERIMENTAL DETAILS

### Text S3.1. PCR protocol.

Duplicate PCR reactions were conducted in a 20 µL volumes using 17 µLof AccuPrime Pfx SuperMix (Life Technologies), 1 µLof genomic DNA (20 ng template), and 1 µL each of the forward and reverse primers (10 µM). The PCR reactions were carried out in a BioRad T100 thermocycler using the following protocol: 5 min initial denaturation at 95°C; 30 cycles of denaturation (95°C, 20 s), annealing (50°C, 15 s) and elongation (68°C, 60 s); and finally 10 min elongation at 68°C. Purification of PCR products was done with the MagJET NGS Cleanup and Size Selection Kit (Thermo Scientific). The DNA concentrations were measured using a Qubit 3.0 (Invitrogen) and the dsDNA HS assay kit (Invitrogen). The PCR products were pooled and sequencing was carried out on an Illumina MiSeq using reagent kit v3.

**Table S3.1.** Accession number (European Nucleotide Archive) and identity of sequence read fastq files used in the analysis of the AGS data set.

| Run accession | Sample type |
|---|---|
| ERR3721823 | R1 |
| ERR3721824 | R1 |
| ERR3721825 | R1 |
| ERR3721826 | R1 |
| ERR3721827 | R1 |
| ERR3721828 | R1 |
| ERR3721829 | R2 |
| ERR3721830 | R2 |
| ERR3721831 | R2 |
| ERR3721832 | R2 |
| ERR3721833 | R2 |
| ERR3721834 | R2 |
| ERR3721835 | Inoculum |
| ERR3721836 | Inoculum |
| ERR3721837 | Inoculum |
| ERR3721838 | Inoculum |
| ERR3721839 | Inoculum |
| ERR3721840 | Inoculum |

**Table S3.2.** Accession number (European Nucleotide Archive) and identity of sequence reads fastq files used in the analysis of the MFC data set. MFC A, B, C, and D refers to separate microbial fuel cells.

| Run accession | Sample type |
|---|---|
| ERR2586221 | Acetate-fed anode MFC A |
| ERR2586222 | Acetate-fed non-conductive biofilm MFC A |
| ERR2586224 | Acetate-fed anode MFC B community replicate 1 |
| ERR2586225 | Acetate-fed anode MFC B community replicate 2 |
| ERR2586226 | Acetate-fed anode MFC B community replicate 3 |
| ERR2586227 | Acetate-fed anode MFC B community replicate 4 |
| ERR2586228 | Acetate-fed anode MFC B community replicate 5 |
| ERR2586229 | Acetate-fed anode MFC B community replicate 6 |
| ERR2586230 | Acetate-fed non-conductive biofilm MFC B community replicate 1 |
| ERR2586231 | Acetate-fed non-conductive biofilm MFC B community replicate 2 |
| ERR2586232 | Acetate-fed non-conductive biofilm MFC B community replicate 3 |
| ERR2586233 | Acetate-fed non-conductive biofilm MFC B community replicate 4 |
| ERR2586234 | Acetate-fed non-conductive biofilm MFC B community replicate 5 |
| ERR2586235 | Acetate-fed non-conductive biofilm MFC B community replicate 6 |
| ERR2586237 | Glucose-fed anode MFC C |
| ERR2586238 | Glucose-fed non-conductive biofilm MFC C |
| ERR2586240 | Glucose-fed anode MFC D community replicate 1, technical replicate 1 |
| ERR2586241 | Glucose-fed anode MFC D community replicate 2 |
| ERR2586242 | Glucose-fed anode MFC D community replicate 3 |
| ERR2586243 | Glucose-fed anode MFC D community replicate 4 |
| ERR2586244 | Glucose-fed anode MFC D community replicate 5 |
| ERR2586245 | Glucose-fed anode MFC D community replicate 6 |
| ERR2586246 | Glucose-fed non-conductive biofilm MFC D community replicate 1 |
| ERR2586247 | Glucose-fed non-conductive biofilm MFC D community replicate 2 |
| ERR2586248 | Glucose-fed non-conductive biofilm MFC D community replicate 3 |
| ERR2586249 | Glucose-fed non-conductive biofilm MFC D community replicate 4 |
| ERR2586250 | Glucose-fed non-conductive biofilm MFC D community replicate 5 |
| ERR2586251 | Glucose-fed non-conductive biofilm MFC D community replicate 6 |
| ERR2586253 | Inoculum (activated sludge) |
| ERR2586257 | Glucose-fed anode MFC D community replicate 1, technical replicate 2 |
| ERR2586258 | Glucose-fed anode MFC D community replicate 1, technical replicate 3 |
| ERR2586259 | Glucose-fed anode MFC D community replicate 1, technical replicate 4 |
| ERR2586260 | Glucose-fed anode MFC D community replicate 1, technical replicate 5 |
| ERR2586261 | Glucose-fed anode MFC D community replicate 1, technical replicate 6 |

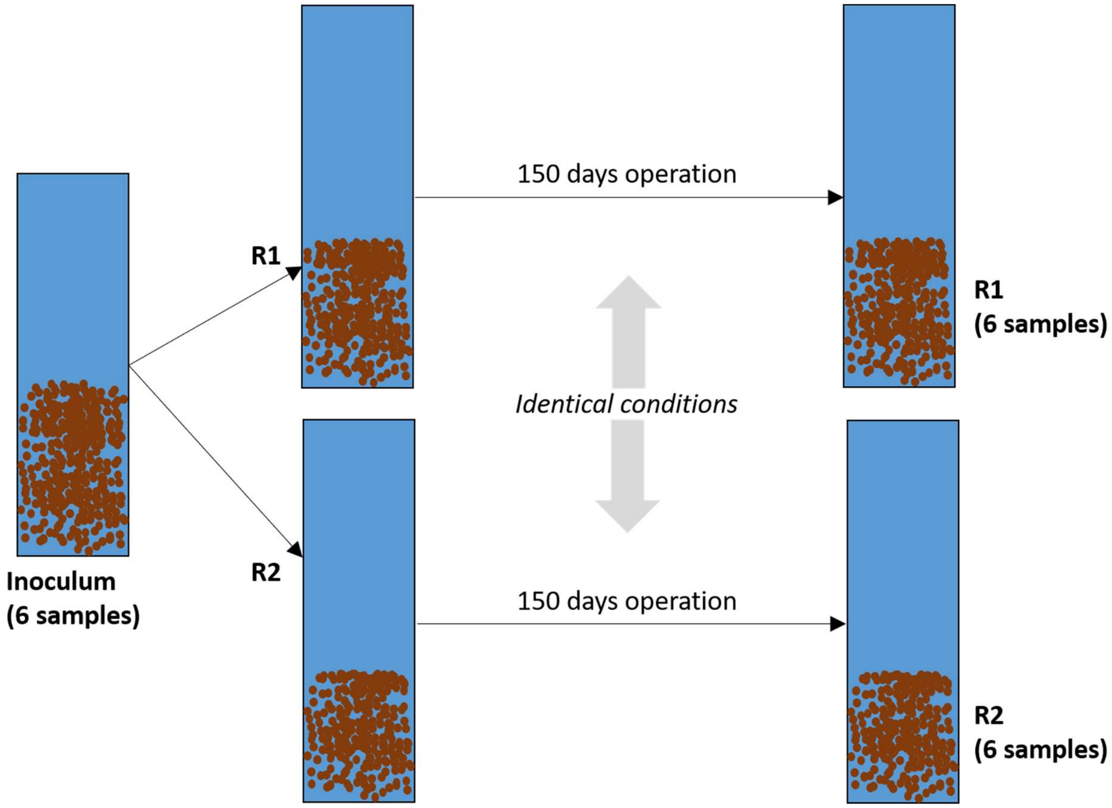


**Figure S3.1.** Schematic of the aerobic granular sludge (AGS) experiment. The sludge in a sequencing batch reactor was used to inoculate two parallel reactors (R1 and R2), which were operated under identical conditions. Six samples were collected from the inoculum and from R1 and R2 after 150 days.

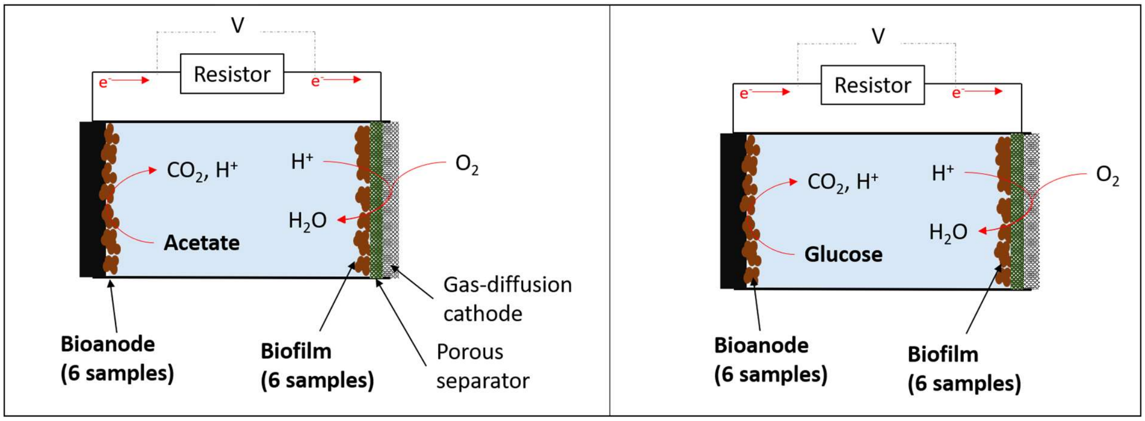


**Figure S3.2.** Schematic of the microbial fuel cell (MFC) setup. Samples were collected from the bioanode and the biofilm covering the porous separator near the gas-diffusion cathode. Samples were collected from both an MFC fed with acetate and one fed with glucose.
